# Comprehensive summary: the role of PBX1 in development and cancers

**DOI:** 10.3389/fcell.2024.1442052

**Published:** 2024-07-26

**Authors:** Mingsheng Liu, Yan Xing, Jiufeng Tan, Xiaoliang Chen, Yaming Xue, Licheng Qu, Jianchao Ma, Xuefei Jin

**Affiliations:** ^1^ 2nd Inpatient Area of Urology Department, China-Japan Union Hospital, Jilin University, Changchun, China; ^2^ Jinlin Provincial Key Laboratory of Molecular Diagnosis of Urological Tumors, Changchun, China; ^3^ Jinlin Provincial Key Laboratory of Urological Tumors, Changchun, China

**Keywords:** PBX1, TALE homeodomain protein, E2A-PBX1, development, cancer

## Abstract

PBX1 is a transcription factor that can promote the occurrence of various tumors and play a reg-ulatory role in tumor growth, metastasis, invasion, and drug resistance. Furthermore, a variant generated by fusion of E2A and PBX1, E2A-PBX1, has been found in 25% of patients with childhood acute lymphoblastic leukemia. Thus, PBX1 is a potential therapeutic target for many cancers. Here, we describe the structure of PBX1 and E2A-PBX1 as well as the molecular mecha-nisms whereby these proteins promote tumorigenesis to provide future research directions for developing new treatments. We show that PBX1 and E2A-PBX1 induce the development of highly malignant and difficult-to-treat solid and blood tumors. The development of specific drugs against their targets may be a good therapeutic strategy for PBX1-related cancers. Furthermore, we strongly recommend E2A-PBX1 as one of the genes for prenatal screening to reduce the incidence of childhood hematological malignancies.

## 1 Introduction

Transcription factors (TFs) are a class of proteins that regulate the temporal and spatial expression of target genes in different cells. These proteins serve as administrators of the genome, shaping the phenotype of the organism by modulating cell fate (Francois et al., 2020). TFs recognize specific DNA sequences to control the local assembly of larger protein complexes, inducing or repressing the transcription of nearby genes. TFs determine when, where, and how the gene product is produced in response to upstream signals (Reichlmeir et al., 2021; Rothenberg, 2022). The number of TFs in human is ∼1,600. Homeodomain TFs play irreplaceable roles in embryogenesis and differentiation. They have a homology domain that is approximately 60 amino acids long and characterized by the inclusion of a three amino acid loop extension (TALE) domain between the first and second α-helices. Homeodomain TFs comprise two families, PBC (PBX1–4) and MEINOX, which are further divided into MEIS and PREP subfamilies (Blasi et al., 2017; Bobola and Sagerström, 2024). Among them, pre-B-cell leukemia homeobox 1 (PBX1) has become the focus of related research. The variant of PBX1, E2A-PBX1, which is generated by the fusion of E2A and PBX1 in the process of chromosomal translocation, is associated with pre-B-cell acute lymphoblastic leukemia (ALL) (Izraeli et al., 1992; Izraeli et al., 1993; Nagel and Meyer, 2022; Mostufi-Zadeh-Haghighi et al., 2023; Sinclair et al., 2023). The network in the process of tumorigenesis induced by E2A-PBX1 is complex, involving a variety of target genes and interacting proteins. This indicates that PBX1 has an important role in tumors and makes it a potential therapeutic target. Here, our aim was to highlight the specific aspects that require further investigation in this field to promote research exploring new treatment options for PBX1-related tumors. To that aim, this review describes the structures of PBX1 and E2A-PBX1 as well as the regulatory networks through which these TFs promote tumorigenesis.

## 2 Review

### 2.1 Structure of PBX1

**FIGURE 1:**
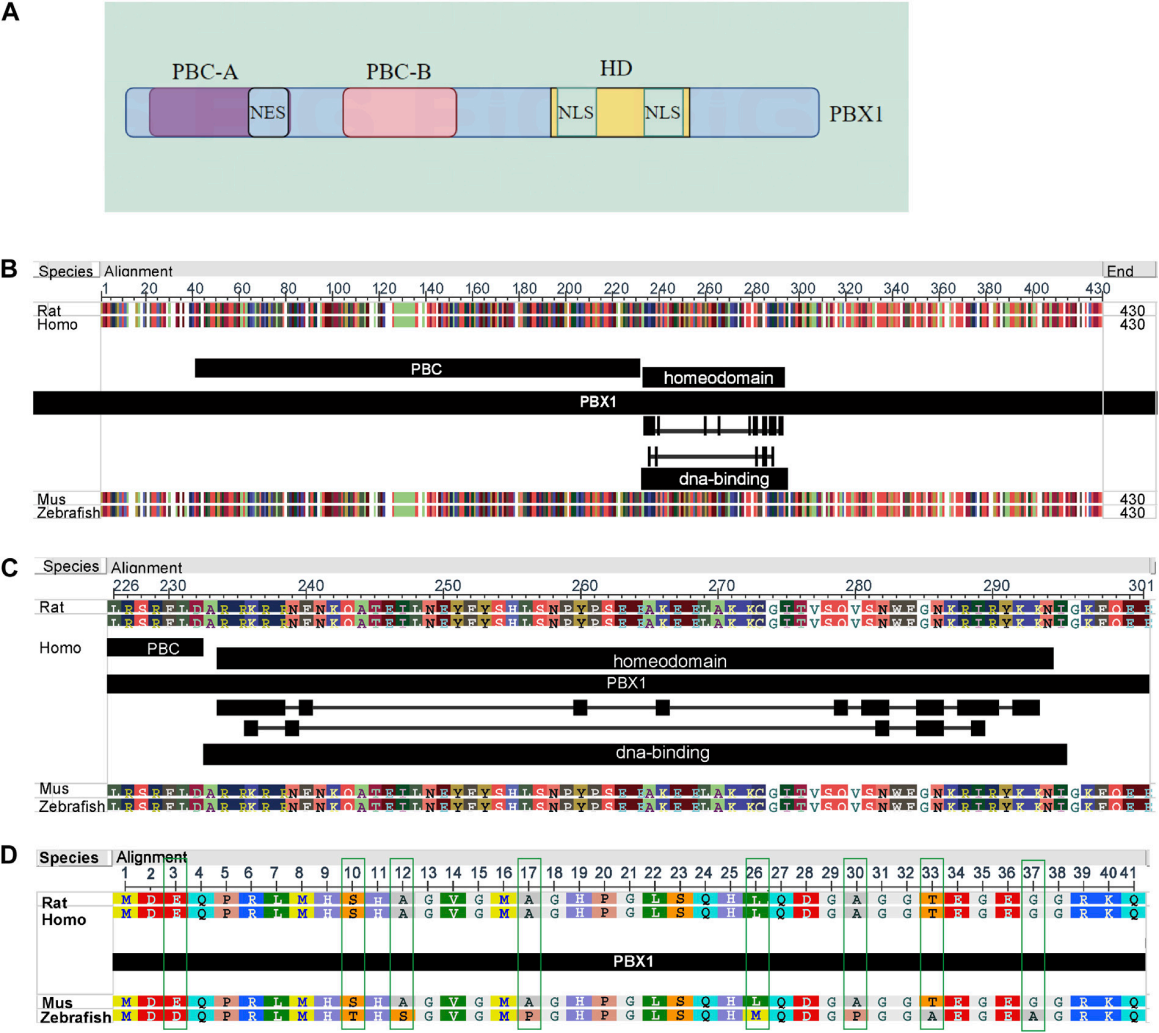
Structure of PBX1and amino acid sequences in different species. **(A)** PBX1 primarily consists of three domains: the PBC-A and PBC-B domains for interacting with other proteins, and the homeodomain (HD). The PBC-A domain contains a nuclear export signal (NES) structure; the PBC-B domain is composed of helices and random coils, functioning as a switch that controls the nuclear import and export of PBX1; the HD domain includes two nuclear localization signals (NLS) regions responsible for the translocation of PBX1 into the nucleus and its positioning on DNA. (Image B was generated by figdraw). **(B–D)** In humans, rats, mice, and zebrafish, the PBX1 protein consists of 430 amino acids, and its structure shows high conservation across different species. Among these four species, variations occur only at positions 3, 10, 12, 17, 26, 30, 33, and 37 in zebrafish. However, in the homeodomain region (positions 232–293) and other regions, the amino acid sequences are completely identical across all four species.

PBX1 was first identified as the fusion partner of E2A in the t(1:19) chromosomal translocation (Kamps et al., 1990; Veiga et al., 2021), which functions as a HOX-cofactor in *D. melanogaster*; flies with mutant PBC protein display similar homeotic transformations to those observed in *HOX*-mutant animals, without remodeling the expression of the relevant HOX genes (Penkov et al., 2013; Grebbin and Schulte, 2017; Gulotta et al., 2021; Olatoke et al., 2023). Lu and De Kumar et al.’s researches excellently elucidated the interaction regions between PBX1, HOX, and DNA (Lu and Kamps, 1996; De Kumar and Darland, 2021). PBX1 is about 430 residues long and, starting from the N-terminus, contains PBC-A and B domains, which are the sites through which PBX1 binds to other proteins to carry out its function, and the highly conserved homeodomain (HD), a DNA binding motif (Longobardi et al., 2014; Bruckmann et al., 2020). The conserved regions of PBC-A (with a nuclear export signal (NES) and PBC-B are composed of helices, whereas the non-conserved areas are composed of random coils. The region between PBC-A and PBC-B contains an alanine-rich stretch of low complexity. This region has been suggested to function as a flexible linker in complex formation with other proteins (Mathiasen et al., 2015; Oriente et al., 2018; Kao et al., 2024). The switch between nuclear PBX1 export and import is controlled by the PBC-B domain, which contains some conserved serine residues that correspond to Ser/Thr kinase phosphorylation sites. With dephosphorylation of the serine residues, PBX1 transports to the cytoplasm (Kilstrup-Nielsen et al., 2003). The HD domain contains nuclear localization signals (NLS) that guide PBX1 transport to the nucleus and binding to the DNA sequence; depletion of the HD domain impairs the binding between PBX1 and DNA (Farber and Mittermaier, 2011; Alankarage et al., 2020; Bruckmann et al., 2020) (Figure 1A). When PBX1 is free in the cytoplasm, the N-terminal and HD regions are tightly connected, preventing PBX1 from entering the nucleus. PBX1 protein structure in different species to is conserved. We downloaded the PBX1 protein sequences of human (ID: P40424), rat (ID: A0A8I5ZR45), mouse (ID: P41778), and zebrafish (ID: H1A3Y0) from UniProt (https://www.uniprot.org/) and analyzed these sequences using the NCBI Multiple Sequence Alignment Viewer 1.25.1 on the NCBI website. The analysis results showed that in humans, rats, mice, and zebrafish, the PBX1 protein consists of 430 amino acids, and its structure shows high conservation across different species. Among these four species, variations occur only at positions 3, 10, 12, 17, 26, 30, 33, and 37 in zebrafish. However, in the homeodomain region (positions 232–293) and other regions, the amino acid sequences are completely identical across all four species (Figures 1B–D). The results suggested that PBX1 is highly conserved across different species.

### 2.2 PBX1 in development

**FIGURE 2:**
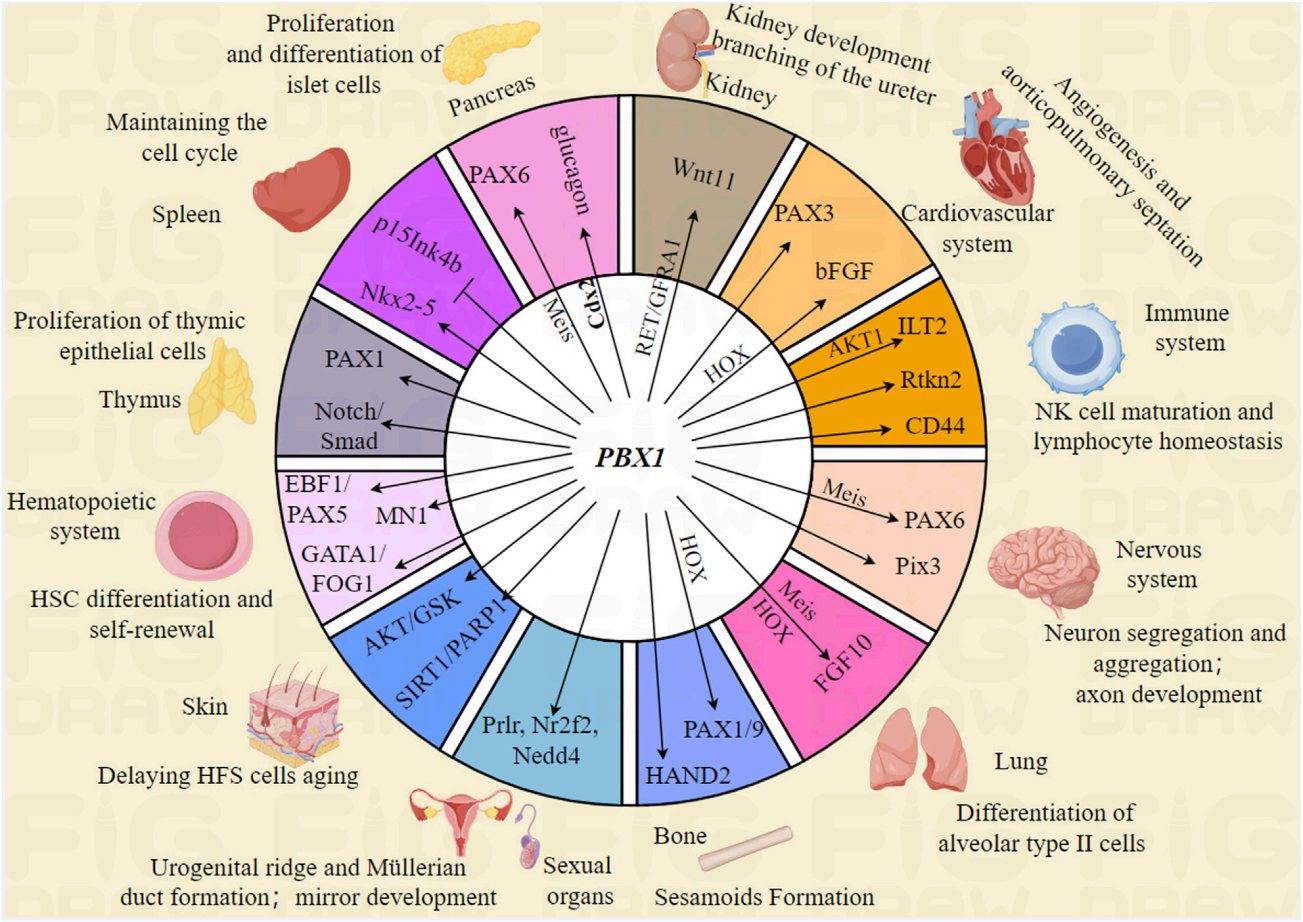
PBX1 maintains the normal development and functional integrity of the organism. PBX1 plays crucial roles in various biological processes by regulating target genes, including hematopoietic system development, thymus formation, spleen development, pancreatic development, cardiovascular system regulation, kidney development, immune system function, nervous system organization, lung development, bone formation, sexual organ development, and skin integrity. Its absence or mutation can lead to significant developmental defects and impair normal physiological functions across multiple organ systems. (Images were designed and generated by figdraw).

In the research history of PBX1, PBX1 has been demonstrated to play a role in the regulation of nearly all body organs and tissues during development (Figure 2).

#### 2.2.1 Hematopoietic system

PBX1 has been reported to be expressed in hematopoietic progenitor cells during mouse embryonic development. It was reported that deletion of the PBX1 gene in mouse embryos caused embryonic death in embryos day 15 (E15) or E16 with handicapped hematopoiesis (Kim et al., 2002; Ficara et al., 2013). Specifically, in PBX1-knockout embryos, hematopoietic lineages may experience developmental defects, leading to impaired hematopoietic function. These defects may include a decrease in the number of hematopoietic progenitor cells and myeloid cells, as well as defects in the differentiation and maturation of erythrocytes and megakaryocytes. These issues may result in severe hematopoietic defects during embryonic development, leading to embryonic death (Ariki et al., 2014; Dutta et al., 2021; Zhang K. et al., 2023). PBX1 supports HSC self-renewal, and transcriptional analysis reveals that multiple stem cell maintenance programs are disrupted in the absence of PBX1 (Ficara et al., 2008; Crisafulli et al., 2019; Dutta et al., 2021). Although PBX1 is crucial for HSPC differentiation, the generation of myeloid and erythroid lineages might be compensated by other transcription factors and signaling pathways. Myeloid and erythroid cells might rely on different developmental pathways that involve other critical regulatory factors in their regulatory networks, allowing them to be generated even without PBX1. For instance, transcription factors like RUNX1 and GATA2(GATA-binding transcription factor 2) play key roles in the generation of myeloid and erythroid cells and can partially compensate for the absence of PBX1. PBX1 is indispensable in the differentiation of hematopoietic stem cells, and the ineffective PBX1 leads to impairment of B cell development and megakaryocyte generation (Okada et al., 2003; Ficara et al., 2013; Zewdu et al., 2016; Cullmann et al., 2021). To be specific, PBX1 regulates various genes related to B cell or megakaryocyte lineage commitment and maturation, including EBF1 (Early B-cell Factor 1) and PAX5 (Paired Box 5) or GATA1(GATA-binding transcription factor 1) and FOG1(Forkhead box G1). These genes are critical for the expansion and maturation of B cell progenitors or megakaryocytes. PBX1 works in coordination with other transcription factors (such as members of the HOX family) to form a complex transcriptional network that ensures the correct formation and function of them. The absence of PBX1 leads to downregulation of these key genes, disrupting the transcriptional programs necessary for B cell and megakaryocytes development, resulting in impaired generation (Familiades et al., 2009; Gregory et al., 2010; Cullmann et al., 2021; Lee et al., 2021). In addition, it was confirmed that both MN1(Meningioma 1 proto-oncogene) wild-type and mutant proteins copurified with PBX1, which indicated that PBX1 participates in the transcriptional regulation of target genes by interacting with MN1 to regulate body homeostasis such as HSC maintenance and self-renewal (Xu et al., 2018; Miyake et al., 2020). Conditional loss of PBX1 in specific hematopoietic lineages using the Cre-lox system provides valuable insights into the role of PBX1 in these lineages (DiMartino et al., 2001; Fischbach et al., 2005; Sanyal et al., 2007; Grebbin and Schulte, 2017) (Table 1).

**TABLE 1:** Conditional loss of PBX1 in specific hematopoietic lineages using the Cre-lox system.

Lineage	Model	Findings	Mechanistic Insights
B Cell Lineage	CD19-Cre PBX1fl/fl	Significant reduction in B cell numbers Impaired B cell maturation	PBX1 regulates key genes such as EBF1 and PAX5 Disruption in these genes’ regulation leads to impaired B cell development
Megakaryocyte Lineage	PF4-Cre; PBX1fl/fl	Defects in megakaryocyte maturation. Reduced platelet production	PBX1 influences the expression of GATA1 and FOG1 Deletion of PBX1 results in disrupted megakaryocyte differentiation and function
Myeloid and Erythroid Lineages	Mx1-Cre; PBX1fl/fl Vav1-Cre; PBX1fl/fl	Myeloid and erythroid cells can still develop Differentiation and function are not entirely normal	Other transcription factors (e.g., RUNX1 and GATA2) may partially compensate for PBX1 loss PBX1 contributes to the fine-tuning of these differentiation pathways, but its absence leads to suboptimal cell function

#### 2.2.2 Thymus and spleen

PBX1 drives the proliferation of thymocytes (Penkov et al., 2008), and it was reported that *pbx1* homozygous mutants display delayed or absent formation of the caudal pharyngeal pouch in mice, as well as a pattern of disturbance of the third pharyngeal pouch. In addition, in the study of Dimuthu Alankarage, it was found that mutations in PBX1 lead to ectopic or absent thymus and spleen in mice (Brendolan et al., 2005; Zewdu et al., 2016; Alankarage et al., 2020). Intrinsically, PBX1 is essential for the proliferation of thymocytes. It interacts with transcription factors such as PAX1, regulating their expression and promoting thymocyte proliferation (Danso-Abeam et al., 2013; Selleri et al., 2019). PBX1 also regulates thymocyte differentiation and maturation through interaction with genes like SMAD and Notch, which are critical for the differentiation and maturation process. Additionally, PBX1 controls thymocyte fate by modulating the expression of these and other related genes, thereby determining the destiny of thymic hematopoietic cells (Jurberg et al., 2015; McCarron et al., 2019; Mary et al., 2023). Mice with mesenchymal-specific PBX1 inactivation in the spleen exhibit hyposplenism which may be related to the downregulation of Nkx2-5 and the upregulation of p15Ink4b caused by *pbx1* deletion in spleen mesenchymal progenitor cells which resulted in cell cycle disorder (Brendolan et al., 2005; Koss et al., 2012).

Extrinsically, PBX1 impacts the thymic microenvironment by regulating stromal cells and other microenvironmental components (Gao et al., 2022). It influences the signaling pathways within these cells, affecting the overall support provided to the cells. PBX1’s role in the stromal microenvironment includes modulating intercellular signaling factors like BMP and Wnt, which are crucial for thymocyte and splenic cell development and function (Figueiredo et al., 2020; Khumukcham and Manavathi, 2021; Arnovitz et al., 2022).

#### 2.2.3 Pancreas

PBX1 is crucial during the early stages of pancreatic development. PBX1 is involved in the morphogenesis of the pancreas, influencing the branching and formation of pancreatic ducts and acinar structures. It participates in the specification and patterning of the pancreatic progenitor cells, and helps establish the domains where the pancreas will form within the developing embryo (Kim et al., 2002; Oriente et al., 2018). It was reported that PBX1 in the pancreas promotes the proliferation and differentiation of stem cells, and PBX1 enhances the expansion of stem cells, which leads to the regeneration of islet cells from ductal and potentially acinar cells in a rat pancreatic ligation model (Peters et al., 2005). In adult pancreatic cells, PBX1 continues to play a significant role in maintaining islet cell function and cellular homeostasis. It helps regulate the expression of genes necessary for the normal function of pancreatic cells. In the research of Xin Zhang, the authors provided biochemical and transgenic data showing that the key islet development regulation process is achieved by PBX1 through its action on PAX6 (Zhang et al., 2006). In addition, studies have shown that PBX1 is involved in the regulation of proglucagon gene expression by regulating caudal-related homeobox transcription factor 2 (Cdx2) and ensures the expression of glucagon (Herzig et al., 2000; Liu et al., 2006).

PBX1 is particularly important in maintaining the function of pancreatic beta cells, which produce insulin. It ensures the proper expression of insulin and other hormones essential for glucose homeostasis (Kim and MacDonald, 2002; Pan and Wright, 2011; Dai et al., 2021). It promotes the reprogramming of adipose-derived mesenchymal stem cells into islet β cells, aiding in the directed differentiation and functional maintenance of these cells (Dai et al., 2022). This activity is particularly relevant for therapeutic applications, such as in the treatment of diabetes, where reprogrammed cells help in lowering blood sugar levels in diabetic models (Oriente et al., 2018; Engwa et al., 2020). PBX1 is involved in the response to pancreatic injury and regeneration. In cases of damage or disease, such as in diabetes or pancreatitis, PBX1 helps orchestrate the repair processes, although its exact mechanisms and effects can vary (Scarlett et al., 2011).

#### 2.2.4 Cardiovascular system

PBX1 coordinates with transcriptional pathways to control aortic patterning and cardiac outflow tract (OFT) compartmentation in mice and *pbx1*-null embryos display abnormal aorta and OFT septation failure. PBX1 regulates PAX3 expression to control the development of the OFT in the heart. The DNA sequence elements of PAX3 contain consensus binding elements for PBX heterodimers and have been shown to interact with members of the PBX family. In *pbx1*-deficient embryos, a brief burst of PAX3 expression in premigratory cardiac neural crest cells (NCC) prior to migration is lost, ultimately determining the function of cardiac NCCs in promoting OFT development. Disruption of the PBX1-PAX3 regulatory pathway is partly responsible for the OFT deficiency observed in *pbx1*-deficient mice (Pruitt et al., 2004; Chang et al., 2008). In a clinical study, it was found that about 33% of the patients’ families were associated with *pbx1* mutations in the genetic analysis of patients with congenital heart disease (Szot et al., 2018). Similarly, Anne Slavotinek described 8 patients who were heterozygous for the *pbx1* variant and found that mutations in this gene cause malformations of heart (Slavotinek et al., 2017). Similar results were found in the study by Dimuthu Alankarage, where mutations in PBX1 resulted in the absence of the pulmonary valve (Alankarage et al., 2020). Endothelial cells (EC) is the source of mesenchymal cells in the middle of the endocardial pad, which form the structural elements of the atrioventricular valve as well as the atria and membranous interventricular septum (Miao et al., 2020). PBX1 is essential for proangiogenic HOX DNA binding and transcriptional activity in EC. Increased expression of PBX1 in ECs facilitates more efficient complex formation on PBX1/HOX consensus DNA oligonucleotides. Conversely, mutations in PBX1 impair the binding of the PBX1/HOX complex to target DNA, as well as EC migration and bFGF-induced angiogenesis *in vivo* (Charboneau et al., 2005).

#### 2.2.5 Kidney

Pauline Le Tanno reported a cohort of 8 patients with *pbx1* deletion. All patients presented with congenital renal and urinary tract malformations, mainly bilateral renal hypoplasia, with or without dysplasia (Le Tanno et al., 2017). The same findings were also observed in the research of Friederike Petzold and Laurence Heidet through whole genome sequencing (Heidet et al., 2017; Petzold et al., 2022), which coincides with the research results of Ling Nie (Nie et al., 2022). It was reported that PBX1 regulates the development of the kidney as well as the branching of the ureter, while deletion of *pbx1* results in a progressive reduction in kidney growth and differentiation. Mechanistically, misexpression of c-Ret oncogene by which PBX1 regulates mesenchymal-epithelial-induced interactions may be correlated with impaired ureteric development in *pbx1* ^−/−^ kidneys (Schnabel et al., 2003a; Hu et al., 2023). Ret regulates cell cycle progression through Growth Factor Receptor-α 1(Gfra1) and Wnt11. The inactivation of Ret leads to a reduction in cell proliferation and alterations in cell cycle-related gene expression. Ret signaling has been shown to regulate Wnt11 expression, which maintains the normal expression level of glial cell line–derived neurotrophic factor (GDNF), ensuring normal outgrowth and branching of ureteric bud cells. GDNF-RET/GFRA1 dysfunction can impact cell proliferation, migration, differentiation, survival, and regeneration (Al-Shamsi et al., 2022; Zhao et al., 2023).

#### 2.2.6 Immune system

PBX1 plays a critical role in the immune system by regulating pathways and transcriptional activities essential for the maturation and function of various immune cells. Its involvement in both promoting immune responses and maintaining immune tolerance highlights its importance in both normal immune function and autoimmune disease contexts. In dNK cells, PBX1 promotes dNK cell maturation and furtherly promotes fetal development. PBX1 upregulates the AKT1 pathway which activated by the interaction of major histocompatibility complex G with the immunoglobulin-like transcript 2 receptor (ILT2) and drives transcription of pleiotropin and osteoglycine in dNK cells (Zhou et al., 2020). The abnormal development of T cells and B cells caused by the abnormal expression of PBX1 is related to the occurrence of systemic lupus erythematosus. To be specific, PBX1 upholds the equilibrium and constancy of T reg cells via the advancement of the cell cycle by targeting rhotekin 2 (Rtkn2), thus impeding the proliferation of inflammatory T cells that, if left unchecked, could intensify the progression of lupus in the hosts (Gu et al., 2023; Choi et al., 2024). Furthermore, PBX1 regulates the activation of T cell by directly targeting the promoter of CD44 which results in the production of self-reactive activated CD4^+^T cells (Oriente et al., 2013; Niu et al., 2017). In lupus murine model with B cell-specific deletion of *pbx1*, the downregulation of PBX1 in autoimmune B cells has been linked to the exacerbation of systemic lupus erythematosus (SLE). The deficiency of *pbx1* in B cells leads to an exaggerated humoral response upon immunization. SLE mice with B cell-specific *pbx1* deficiency exhibit enhanced germinal center responses, increased plasma cell differentiation, and elevated autoantibody production. Consequently, researchers suggested that PBX1 could be targeted as a potential therapeutic strategy for SLE.

#### 2.2.7 Nervous system

PBX1 is essential for the proper formation of various neural structures, including the brain and spinal cord. Its expression patterns during embryogenesis are tightly regulated, ensuring the correct spatial and temporal development of the nervous system (Selleri et al., 2004). In the mature nervous system, PBX1 continues to be important for maintaining neural function. It regulates genes, such as HOX, MEIS2, PAX6 and Paired-like homeodomain transcription factor 3 (Pitx3), involved in neurotransmitter synthesis, neural connectivity, and synaptic plasticity. By modulating the expression of these genes, PBX1 helps sustain normal neural activity and cognitive functions. Additionally, PBX1 is implicated in the response to neural injury and repair processes, highlighting its role in neural health and resilience (Capellini et al., 2006; Grebbin et al., 2016; Hanley et al., 2016; Villaescusa et al., 2016). In addition, by finely tuning the expression of DCC, PBX1 fosters the development and maturation of axons in dopaminergic neurons, a mechanism that could have profound implications for the normal physiological functions of the nigro-striatal system (Sgadò et al., 2012). DCC is an extracellular domain composed of more than 1,000 amino acids, which can promote axonal chemotaxis when combined with Netrin-1 (Wang K. et al., 2020). The study has unveiled several conserved PBX1 binding sites within the intron of the DCC gene, strongly suggesting that PBX1 may directly regulate DCC transcription.

#### 2.2.8 Lung

During embryogenesis, PBX1 is expressed in the developing lung tissue, where it regulates the proliferation and differentiation of lung progenitor cells. This regulation ensures the proper formation of the bronchial tree and alveoli, which are essential for effective respiratory function. PBX1 is involved in the regulation of the normal development of pulmonary blood vessels. Deletion of Pbx gene leads to the misexpression of vasoconstrictor and vasodilator in multiple pathways, which together increase the phosphorylation of myosin in vascular smooth muscle (VSM) cells, thus causing persistent contraction and resulting in the failure of VSM relaxation after birth. The specific mechanism is not very clear, but new evidence suggests that PBX1 may achieve its regulatory role in pulmonary vascular development by regulating T-Box domain-containing protein 2 (TBX2) (McCulley et al., 2018; Lüdtke et al., 2021). In addition, In the process of lung development, with the mutual support of Meis and HOX proteins, PBX1 binds to the promoter of *Fgf10* directly to enhances *Fgf10* transcription which controls the differentiation of alveolar type II cells in the lung epithelium (Li et al., 2014a).

#### 2.2.9 Bone

PBX1 is expressed in osteoblast precursors and other mesenchymal cells, where it regulates the differentiation and proliferation of these cells. PBX1 ensures the correct formation of bone structures by controlling the expression of genes that govern cell growth, differentiation, and mineralization during embryogenesis (Cheung et al., 2009). The posterior axis of the cranial skeleton consists of a series of vertebral bodies and intervertebral discs and adjacent ribs and sternum (Gordon et al., 2011). PBX1 was found to be highly expressed in the notochords, nodes, and vertebral primordia. *Pbx1* mutant showed significant flattening of all vertebral bodies, laminar thinness, loss of transverse characteristics in all regions of the spine, and the presence of residual ribs. PBX1 may regulate the development of posterior cranial axis bones through PAX1 and PAX9 (Capellini et al., 2008). In process of development of long bone, it was suggested that collaboration with Gli3, HOXA11 and HOXD11, PBX1 was involving in regulating the morphology of long bones (Eyal et al., 2019). In addition, recent research indicated that PBX1 modulates limb phenotypes through binding interactions with cofactors such as HAND2, which confers limb bud functions and the absence of PBX1 may potentially induce the occurrence of limb defects (Losa et al., 2023). However, PBX1 plays a time-dependent role in regulating osteogenic gene expression during osteogenesis. For instance, PBX1 inhibits HOXA10’s ability to activate osteoblast-associated genes, thus establishing temporal gene expression regulation during osteogenesis (Gordon et al., 2010). Nevertheless, the precise regulatory details at specific time points during osteogenesis still need further exploration.

#### 2.2.10 Sexual organs

PBX1 plays a significant role in the differentiation and growth of reproductive systems. In males, PBX1 is essential for the proper formation of the testes and the development of Sertoli and Leydig cells, which are vital for sperm production and hormone secretion, respectively. The normal development of the gonads is predicated on PBX1. In addition, PBX1 is essential for maintaining the function of interstitial cells in the testis, and PBX1 deficiency damages testis structure and causes abnormal spermatogenic tubule organization. PBX1 directly regulates the transcription of genes that play crucial roles in steroidogenesis, such as Prlr, Nr2f2, and Nedd4 (Moisan et al., 2022; Wang FC. et al., 2024). In females, PBX1 contributes to the development of the ovaries and the maturation of ovarian follicles, which are critical for ovulation and hormone production. In females, the absence of PBX1 significantly reduces the growth of the urogenital ridge, leading to the absence of the Müllerian duct, but the mechanism underneath is not clear (Schnabel et al., 2003b; Ma et al., 2011; Kia et al., 2019). Moreover, PBX1 is also crucial for maintaining the normal function of the uterus. PBX1 drives the transcription of pleiotrophin and osteoglycin in uterine natural killer (dNK) cells, furtherly promoting fetal development (Zhou et al., 2020).

#### 2.2.11 Skin

PBX1 has been reported to have low levels in the skin throughout development, with its expression continuing to decrease in adult skin, and most of the signal located in basal cells of the epidermis (Kömüves et al., 2000). Although its expression levels are low, PBX1 is important for skin development and maintaining the structural integrity and function of the skin. In our previous researches, we found that, PBX1 plays a crucial role in promoting the proliferation of hair follicle stem cells and delaying their aging. It enhances the proliferation and reprogramming of hair follicle mesenchymal stem cells by activating the AKT/glycogen synthase kinase signaling pathway (Jiang et al., 2019). In another study, we found that PBX1 may help maintain the stemness of hair follicle stem cells and delay their aging by counteracting ROS-induced oxidative stress and reducing DNA damage by activating SIRT-PARP1 axis (Wang et al., 2021; Wang et al., 2023). In addition, we further verified the role of PBX1 in hair follicle stem cells by preparing TAT-PBX1 fusion protein and acting on hair follicle stem cells *in vitro* (Wang B. et al., 2020).

## 3 PBX1 in tumors

### 3.1 PBX1 fusion protein (E2A-PBX1) in ALL

It has been reported that 25% of children with ALL have a t(1; 19) (q23; p13) chromosomal translocation. This causes the fusion of E2A and PBX1 coding sequences to form E2A-PBX1, and this site-specific fusion is defined as a major pathogenic incident in t(1; 19), which is related to the prognosis (Hunger et al., 1991; Zhang H. et al., 2023; Migita et al., 2023).

**FIGURE 3:**
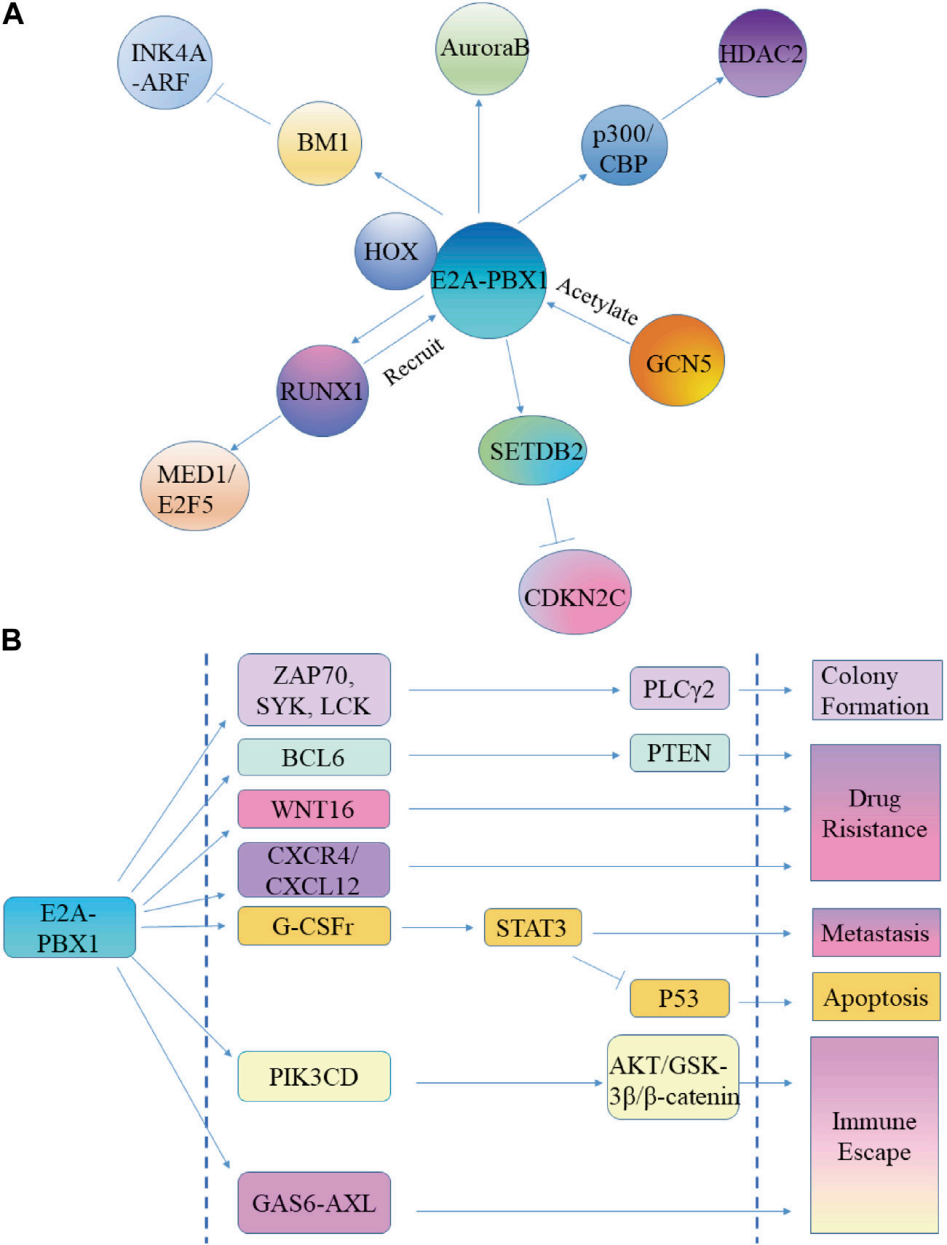
E2A-PBX1 promotes ALL cell proliferation, while increasing its malignancy. **(A)** E2A-PBX1 promotes ALL cell proliferation capacity by regulating downstream genes. **(B)** E2A-PBX1 promotes ALL malignancy by regulating target genes and exerting inhibitory apoptosis, promotional clonogenesis, enhanced migration, increased drug resistance, and immune evasion functions.

E2A protein is a TF that regulates the specific development of cell lineages and is critical in the process of instructing lymphocyte development. E2A encodes E12 and E47 proteins, and each member contains a C-terminal basic helix-loop-helix (bHLH) domain and two activation domains (AD1, AD2) (Aronheim et al., 1993; Belle and Zhuang, 2014). bHLH plays an important role in protein dimerization and identifies the E-box DNA sequence, while the activation domains adjust the transcription of target genes in the presence of cell recruitment cofactors (Murre, 2019; Hao et al., 2021). The E2A-PBX1 protein includes the N-terminal region of E2A, which contains the AD1 and AD2 domains, and most of the PBX1 structure (LeBrun, 2003). Expression of E2A-PBX1 affects the central regulatory path of the hematopoietic process, affecting signaling molecules such as WNT, cell cycle control signals, or apoptosis, which contributes to the development and progression of hematologic malignancies (Diakos et al., 2014) (Figure 3).

The oncogenicity of E2A-PBX1 is AD activation domain-dependent; the helical portion of AD1 directly interacts with the transcriptional coactivator ring AMP response element-binding protein (CBP) to promote immortalization of primary bone marrow cells (Bayly et al., 2006). There is a conserved 17-residue area in AD1, called the PCET motif, which binds to the KIX domain of the transcriptional coactivator CBP/p300, causing epigenetic changes in cells critical for leukemogenesis (Denis et al., 2012; Pi et al., 2020). However, p300/CBP catalyzes the acetylation of H3K18Ac and H3K27Ac histones, and regulates the expression of HDAC2 to change the metabolism of tumor cells and promote their growth (Tang et al., 2019; Cai et al., 2021; Nicosia et al., 2023) (Figure 3A). General control nonderepressible 5 (GCN5) and p300/CBP-related factors are members of the same family. GCN5 acetylates E2A-PBX1 and increases the steadiness of E2A-PBX1 in tumor cells (Holmlund et al., 2013) (Figure 3A).

SETDB2 maintains expression as a direct target gene of the fusion transcription factor E2A-PBX1, and SETDB2 depletion sensitizes E2A-PBX1-positive cell lines to kinases and epigenetic inhibitors (Lin et al., 2018). SETDB2 represses the expression of the cell cycle inhibitor CDKN2C through histone H3K9 trimethylation, establishing an oncogenic pathway (Lin et al., 2018) (Figure 3A).

PBX1 binds to DNA by interacting with cofactors containing TALE-binding domains, including HOX, MEIS, and Prep. Therefore, E2A-PBX1 is also thought to dimerize with other TALE proteins for DNA binding. Together with HOX proteins, it was reported that E2A-PBX1 may activate the transcription of downstream target genes (Pi et al., 2020). However, E2A-PBX1 does not heterodimerize with Meis/PREP1 proteins because fusion to E2a deletes the PBX1 sequence required for cooperative DNA binding with Meis1 (Knoepfler et al., 1997; Calvo et al., 1999) (Figure 3A).

Similar to PBX1, HOX proteins also participate in the functioning of E2A-PBX1. E2A-PBX1 binds to the DNA sequence ATCAATCAA through the PBX1 moiety and in cooperation with HOX proteins such as HOX-A5, HOX-B7, HOX-B8, HOX-C8, and HOX-D4 (Van Dijk et al., 1993; Lu et al., 1995; Pi et al., 2020). Moreover, it binds to HOX through a HOX consensus motif (HCM, a sequence in the PBX1 HD). HCM is crucial for the transcriptional activity and oncogenic potential of E2A-PBX1 in cells, and its deletion or mutation abolishes the ability of E2A-PBX1 to activate the transcription of target genes, indicating that HCM is required for E2A-PBX1-induced tumorigenesis (Chang et al., 1997; Lin et al., 2019). Controversially, however, emerging evidence suggests that the HCM is dispensable for E2A-PBX1 binding to DNA, and that E2A-PBX1 can enhance its DNA-binding stability through oligomer self-association to induce tumorigenesis (Lin et al., 2019).

The role of the PBX1 DNA-binding domain is twofold; on the one hand, it directly binds and activates a specific set of genes; on the other hand, it mediates the interaction with RUNX1. The E2A-PBX1 construct without the PBX1 DNA-binding domain has no transforming activity and is unable to activate the activator or RUNX1 sites through the PBX site (Calvo et al., 1999; Licht, 2020). E2A-PBX1 is recruited to gene-binding sites by RUNX1, and RUNX1 is a target gene of E2A-PBX1, which upregulates the expression of RUNX1 and promotes the transformation and proliferation of leukemia cells. The mechanism is related to the enhancement of the binding of coactivators (p300 and MED1) and the acetylation of H3K27 by E2A-PBX1 and RUNX1, while the binding of E2A-PBX1 to gene enhancers depends on the interaction of RUNX1 (Guo et al., 2012; Pi et al., 2020). MED1 is required for the growth of E2A-PBX1+ leukemia cells. E2F5 is a cell cycle regulator highly expressed in a variety of cancers, while knockdown of E2F5 can interrupt the growth of leukemia cells. RUNX1 recruits E2A-PBX1 to target gene sites, such as E2F5, and cooperates with MED1 to promote the transcriptional activation of E2F5 (Lee et al., 2021; Qi et al., 2021) (Figure 3A).

E2A-PBX1 in B cell progenitors enhances self-renewal, while impeding the differentiation of B cell progenitors, leading to genomic aberrations. E2A-PBX1 is always accompanied by the loss of PAX5 (approximately 44%), which is associated with the activation of the JAK/STAT signaling pathway (Liu et al., 2014; Duque-Afonso et al., 2015). The mutation of PAX5 occurs in 6.9% of E2A-PBX1^+^ B-ALL patients, while an increased copy number of AKT3 occurs in 92%, which is closely related to relapsed and refractory leukemia (Zhou et al., 2021).

BMI-1 is known as a lymphoid oncogene, whose product acts as a transcriptional repressor of the INK4A-ARF tumor suppressor locus (Jacobs et al., 1999; Dhawan et al., 2009). Studies have shown that E2A-PBX1 upregulates the expression of BMI-1, while *Bmi-1*-deficient hematopoietic progenitor cells show resistance to E2A-PBX1-induced tumor metaplasia. In addition, the negative effects of E2A-PBX1 on pre-B cell survival and differentiation are partly evaded by enforced utterance of p16Ink4a. These findings suggest that E2A-PBX1 may promote the transformation of pre-B cells through the downregulation of INK4A-ARF mediated by BMI-1 (Smith et al., 2003) (Figure 3A).

E2A-PBX1 can target angiopoietin 3, which regulates the proliferation of vascular endothelial cells. Mouse angiopoietin 3 also induces events that promote cell proliferation, such as increasing the number of rough endoplasmic reticula, polysomes, and mitochondria in cells (Fu et al., 1999). Apparently, additional angiogenesis is not required in E2A-PBX1-induced ALL. However, whether Angiogenin-3 causes the increase in the number of endoplasmic reticula and mitochondria in ALL cells and provides favorable conditions for the survival of ALL cells should be explored.

The mitotic regulator aurora kinase is closely related to childhood acute leukemia, especially in E2A-PBX1^+^ ALL cases. Inhibition of Aurora B using shRNA leads to proliferation arrest and apoptosis (Hartsink-Segers et al., 2013). Throughout the G2 phase, Aurora B regulates the formation and function of protein complexes, ensures proper connectivity between microtubules and centromeres, checks for the presence of unattached centromeres, and regulates chromatid segregation (Fu et al., 2007). Therefore, E2A-PBX1 may maintain the replication and proliferation of tumor cells by regulating the expression of Aurora B (Figure 3A).

### 3.2 E2A-PBX1 enhances tumor malignancy

E2A-PBX1 exerts its inhibitory apoptosis, promotional clonogenesis, enhanced migration, increased drug resistance, and immune evasion functions by regulating target genes, thus increasing the malignancy of ALL (Figure 3B).

E2A-PBX1 regulates the expression of PIK3CD, which is encoded by p110δ, by binding to a leukocyte-specific promoter, and its expression acts as an oncogenic driver in tumors (Eldfors et al., 2017; Xenou and Papakonstanti, 2020). PIK3CD activates cell growth and migration by activating the AKT/GSK-3β/β-catenin axis in tumor cells, and this may also be one of the reasons why leukemia cancer cells are able to evade immune surveillance (Chen et al., 2019; Wu et al., 2021; Grüninger et al., 2022). E2A-PBX1 attaches to an enhancer element upstream of CXCR4, which regulates the expression of CXCR4 in the cells that possess the chimeric gene. CXCL12, a chemokine generated by bone marrow stromal cells, binds to the receptor CXCR4 on the cell surface to regulate cell activity. CXCR4/CXCL12 mediates the adherence between leukemia cells and stromal cells, which eventually leads to the generation of adhesion-mediated drug resistance. CXCR4 overexpression can only be observed when the tumor relapses, indicating that E2A-PBX1 works together with other synergistic factors to regulate the expression of CXCR4 and enhance the drug resistance of tumor cells (Eldfors et al., 2017; Szekely et al., 2018; Nengroo et al., 2021).

The expression of granulocyte colony-stimulating factor receptor (G-CSFr) is especially increased in E2A-PBX1-expressing pre-B cells (de Lau et al., 1998). However, G-CSF contributes to the proliferation and metastasis of tumor cells by activating STAT3, which directly acts on the corresponding target genes and microRNA genes that control cell differentiation and stemness. In addition, STAT3 antagonizes the effect of p53 through different mechanisms, disrupting the cell cycle and apoptosis (Agarwal et al., 2015; Karagiannidis et al., 2021).

E2A-PBX1 may delay the senescence of tumor cells. Whole-genome sequencing data from paired samples of 653 pediatric patients across 23 cancer types suggested that E2A-PBX1 may lengthen tumor cell telomeres. Telomere shortening leads to increased genomic instability and induced senescence. In cancer cells, activation of telomerase (TERT) can reverse telomere shortening, thereby keeping telomere length and allowing cells to divide permanently (Wang Z. et al., 2020). Whether E2A-PBX1 prolongs the telomeres of tumor cells in this way needs further study.

PLCγ2 is a central enzyme in B cell receptor signaling in developed B cells, and its hyperactivation in E2A-PBX1^+^ leukemia cells plays an important role in leukemogenesis. E2A-PBX1 promotes sustained upregulation of ZAP70, SYK, and LCK, which are located upstream of PLCγ2. Their depletion results in decreased pPLCγ2 expression and reduced colony formation in an E2A-PBX1^+^ALL cell line (Duque-Afonso et al., 2016). Activation of PLCγ and subsequent IP3 generation promotes the release of the calcium accumulated in the ER, which results in activation of calcineurin and various protein kinase C (PKC) isoforms to promote B cell activation (Baba and Kurosaki, 2011; Feske et al., 2012; Dhami et al., 2022). Mature B cells express both SERCA2b and SERCA3-type calcium pumps; however, SERCA3 is downregulated in E2A-PBX1^+^ ALL cells, which can be reversed by PKC activators. E2A-PBX1 may downregulate the expression of SERCA3 and block the differentiation of pre-B cells by inactivating PKC (Aït Ghezali et al., 2017; Chen et al., 2021). The role of PLCγ in E2A-PBX1^+^ and normal B-cell progenitors seems contradictory. The different outcomes may be caused by the hyperphosphorylation of PLCγ in E2A-PBX1, which leads to changes in the cellular metabolic network (Jing et al., 2021).

WNT-16 is generally expressed in peripheral lymphoid organs rather than bone marrow. Interestingly, WNT-16 transcription is very high in the bone marrow and cell lines of E2A-PBX1-positive pre-B ALL patients. However, blocking of E2A-PBX1 expression results in a marked decrease in WNT-16 mRNA levels, which weakens the proliferation of tumor cells (McWhirter et al., 1999; Schwaller, 2006). WNT16 is considered a hidden molecule mediating cisplatin resistance. Increased Wnt16 expression in cancer promotes the generation of drug resistance (Sun et al., 2016; Hu et al., 2017; Zhou et al., 2023). In summary, WNT16 may promote drug resistance in E2A-PBX1^+^ ALL and increase the malignancy of tumors.

Furthermore, E2A-PBX1 upregulates the expression of BCL6, which is present in a subset of B-lymphoblastic leukemias, particularly in the cases covering the t(1; 19) translocation. BCL6 drives malignant phenotypes by regulating target genes involved in cell proliferation, DNA damage perception, and resistance to apoptosis (Deucher et al., 2015; Guo et al., 2021), such as the tumor suppressor gene PTEN. Increased expression of BCL6 further suppresses the transcription of PTEN, which enhances drug resistance of cancer cells (Liu et al., 2022a).

E2A-PBX1-positive ALL cells can interact with other cells to avoid death. Human osteoblasts secrete GAS6, which induces the migration of E2A-PBX1-positive ALL cells. GAS6, a ligand for members of the TAM (TYRO3, AXL, MER) receptor tyrosine kinase family, supports E2A-PBX1-positive ALL cell survival by inducing dormancy and prevents chemotherapy-induced apoptosis (Shiozawa et al., 2010). The GAS6-AXL complex participates in the preservation of cancer cell survival, inducing cytoskeletal rearrangements, facilitating cell spreading and elongation, and driving invasion (Zdżalik-Bielecka et al., 2021; Bellomo et al., 2022). In addition, GAS6-AXL contributes to tumor cell evasion of immune surveillance and secretion of immunosuppressive factors (Tanaka and Siemann, 2020). Therefore, GAS6 may serve as a therapeutic target for E2A-PBX1 tumors.

### 3.3 PBX1 and E2A-PBX1 in other tumors

**FIGURE 4:**
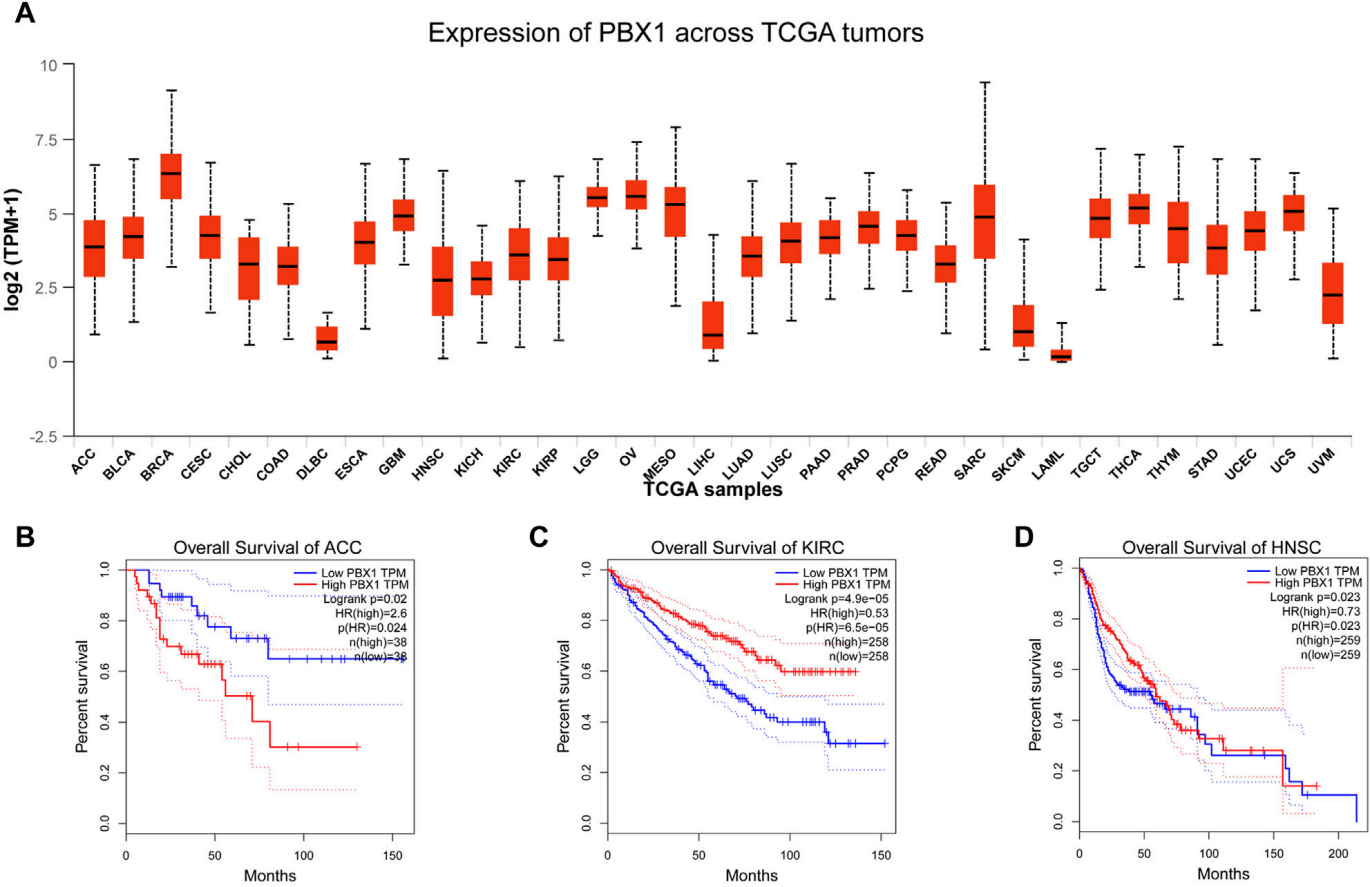
The expression levels of PBX1 in cancers and survival curves of patients with high PBX1 expression. **(A)** The expression level of PBX1 varies among different types of tumors. ACC (Adrenocortical carcinoma), BLCA (Bladder Urothelial Carcinoma), BRCA (Breast invasive carcinoma), CESC (Cervical squamous cell carcinoma and endocervical adenocarcinoma), CHOL (Cholangiocarcinoma), COAD (Colon adenocarcinoma), DLBC (Lymphoid Neoplasm Diffuse Large B-cell Lymphoma), ESCA (Esophageal carcinoma), GBM (Glioblastoma multiforme), HNSC (Head and Neck squamous cell carcinoma), KICH (Kidney Chromophobe), KIRC (Kidney renal clear cell carcinoma), KIRP (Kidney renal papillary cell carcinoma), LGG (Brain Lower Grade Glioma), OV (Ovarian serous cystadenocarcinoma), MESO (Malignant mesothelioma), LIHC (Liver hepatocellular carcinoma), LUAD (Lung adenocarcinoma), LUSC (Lung Squamous cell carcinoma), PAAD (Pancreatic adenocarcinoma), PRAD (Prostate adenocarcinoma), READ (Rectum adenocarcinoma), SARC (Sarcoma), SKCM (Skin Cutaneous melanoma), LAML (Acute myeloid leukemia), TGCT (Testicular germ cell tumors), THCA (Thyroid carcinoma), THYM (Thymoma), STAD (Stomach adenocarcinoma), UCEC (Uterine Corpus Endometrial Carcinoma), UVM (Uveal Melanoma). **(B–D)** High expression of PBX1 is associated with worse survival rates in patients with adrenocortical carcinoma (ACC) (HR(High) = 2.6, p(HR) = 0.024, n(High) = 38, n(Low) = 38), while better survival rates in patients with Kidney renal clear cell carcinoma(KIRC) (HR(High) = 0.53, p(HR) < 0.0001, n(High) = 258, n(Low) = 258) and Head and Neck squamous cell carcinoma (HNSC) (HR(High) = 0.73, p(HR) = 0.023, n(High) = 259, n(Low) = 259).

PBX1, initially identified for its role in childhood acute lymphoblastic leukemia, has since been implicated in the overexpression of various solid tumors, including esophageal cancer, breast cancer, melanoma, adrenocortical carcinoma, prostate cancer, gastric cancer, colorectal cancer, pancreatic cancer et al. (Magnani et al., 2011; Chen et al., 2012; Feng et al., 2015; Magnani et al., 2015; Francis et al., 2021; Shen et al., 2021; Dai et al., 2023; Crisafulli et al., 2024; Kao et al., 2024). Here, we summarized the expression levels of PBX1 in various cancer types (Figure 4A) (data derived from TCGA), and plotted survival curves for patients with high PBX1 expression that exhibited statistically significant differences (Figures 4B–D) (Gene expression profiles were obtained from TCGA. Survival analysis were analyzed via Gene Expression Profiling Interactive Analysis (GEPIA) (http://gepia.cancer-pku.cn)). The results from the survival curves indicate that high PBX1 expression is associated with survival rates in these cancer patients.

E2A-PBX1 may act on T lymphocytes to induce blood disease. A study has found that E2A-PBX1 transgenic mice develop T cell lymphoma at 5 months of age, resulting in their death. This was thought to be related to the insertion of Pmi1. The specific underlying mechanism is not very clear, but other unknown factors must be working together with E2A-PBX1 and Pmi1 to induce T lymphocyte leukemia (Dedera et al., 1993; Feldman et al., 1997). The cooperation between E2A-PBX1 and HOX also has a certain contribution to the induction of T cell leukemia, as T cell leukemia in Hoxb4 compound transgenic animals is more obvious (Bijl et al., 2008). Stem cell factor may be one of the synergistic factors participating in the development of T lymphocyte leukemia. Studies have shown that E2A-PBX1 can induce the rapid proliferation of mouse pro-T cells and acute T lymphocytic leukemia, but this change is stem cell factor-dependent and is accompanied by the loss of the DNA binding function of E2A-PBX1 (Sykes and Kamps, 2004). However, there are few reports on the relationship between E2A-PBX1 and T cell leukemia. Thus, their relationship and molecular mechanism needs to be further explored.

E2A-PBX1 may also play a role in solid tumors. A study on lung cancer showed that, after investigating the expression of E2A-PBX1 in tumor tissues of 184 patients with non-small cell lung cancer, 12.5% of the patients showed the presence of E2A-PBX1, while the positive rate of E2A-PBX1 in the 13 non-small cell lung cancer cell lines reached 23.1%. In addition, smoking is a risk factor for the occurrence of the E2A-PBX1 fusion gene in women (Mo et al., 2013). This suggests that E2A-PBX1 is not restricted to inducing hematological neoplasia, but also plays a role in the occurrence of solid tumors. In addition, E2A-PBX1 may not necessarily appear at birth, but may be produced because of exposure to risk factors.

### 3.4 Potential mechanisms of PBX1 in regulating development of tumors

**FIGURE 5:**
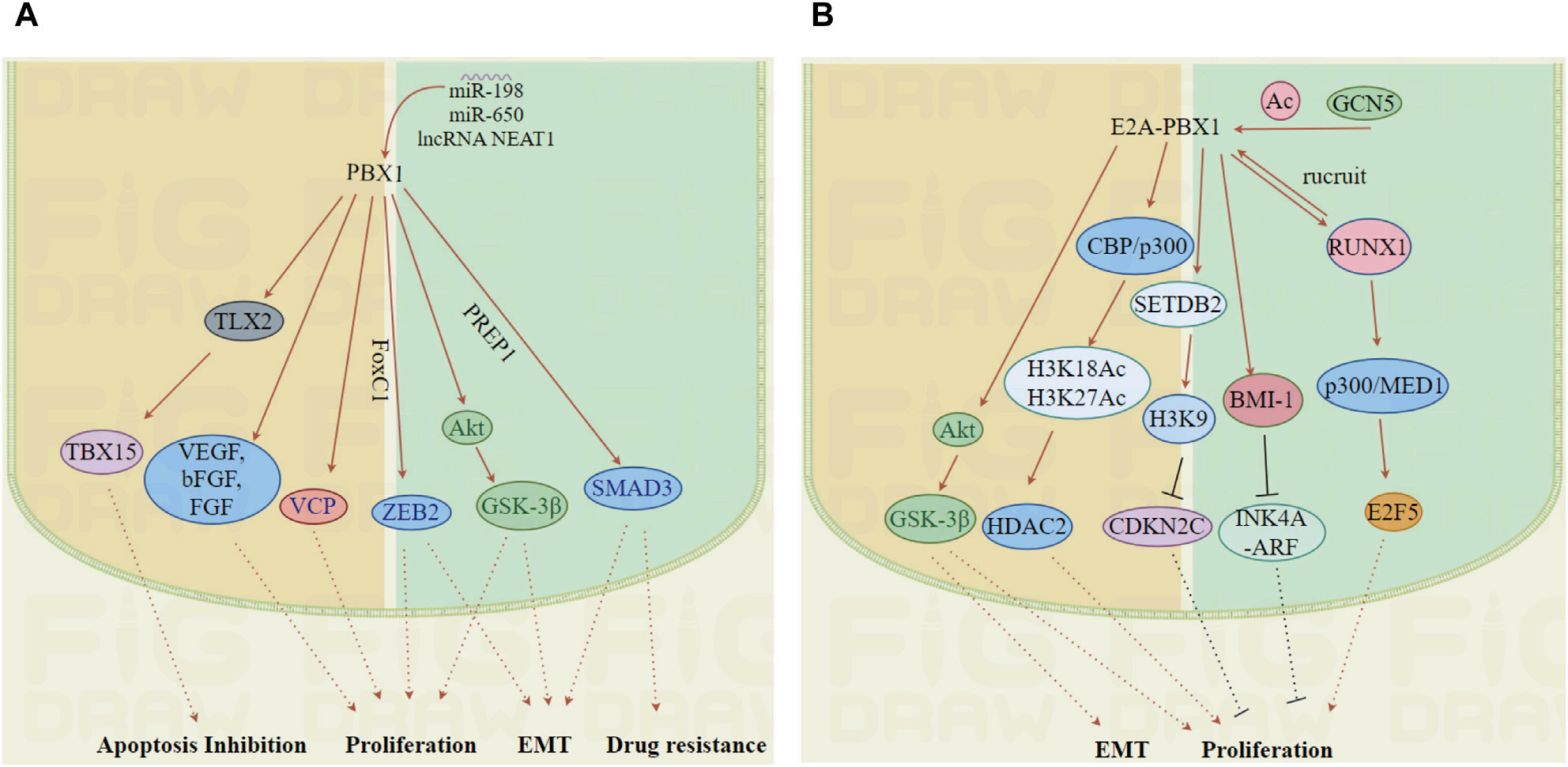
The mechanisms by which PBX1 and E2A-PBX1 promote tumor development. **(A, B)** PBX1 and E2A-PBX1, are associated with the occurrence of multiple tumors and enhance tumor malignancy by affecting multiple pathways including cell cycle regulation, apoptosis inhibition, EMT and drug resistance. (Images were generated by figdraw).

This transcription factor is crucial in the onset and progression of multiple cancers, promoting tumor cell proliferation through interactions with specific proteins or by modulating the transcription of target genes (Figure 5).

For example, PBX1 facilitates the epithelial-mesenchymal transition in esophageal carcinoma by partnering with FoxC1 to recruit it to the ZEB2 promoter. Conversely, suppressing PBX1 enhances the radiosensitivity of esophageal squamous cell carcinoma by targeting STAT3, inhibiting tumor cell proliferation and tumor growth (Zhu et al., 2017; Yu et al., 2020). In myeloproliferative neoplasms, PBX1 influences the stem cell transcriptional program, driving tumor progression (Muggeo et al., 2021). Notably, PBX1 is highly expressed in melanoma cells, and its restorative expression can counteract the growth inhibition mediated by the promyelocytic leukemia zinc-finger protein. Moreover, inactivation of PBX1 by specific small interfering RNA or blockade of the PBX1 promoter region G-4 quadruplex structure by small molecule inhibitors can significantly suppress the growth of melanoma cells. (Shiraishi et al., 2007; Sui et al., 2023). PBX1 also plays a critical role in lung cancer by regulating the expression of invasive transcription factors SMAD3 and Fos-associated antigen 1, involved in metastasis, through its interaction with PREP1. Therefore, developing small molecule inhibitors targeting PBX1 transcriptional signaling could be a novel therapeutic strategy for PBX1-associated cancers (Risolino et al., 2014).

The role of PBX1 in tumor progression varies across different cancer types. In ovarian cancer, PBX1 promotes proliferation and survival through the Notch3 pathway (Park et al., 2008; Fang et al., 2020). While in gastric cancer, it increases tetrahydrobiopterin levels to promote tumor growth and metastasis (Liu et al., 2022b). In renal carcinoma, PBX1 overexpression enhances cell proliferation through the JAK2/STAT3 signaling pathway (Wei et al., 2018).

PBX1 may affect carcinogenesis through its interaction with RNAs. It was found that miR-198 transcription is reduced in pancreatic cancer and that miR-198 inhibition increases PBX-1 and VCP expression; dysregulation of the PBX-1/VCP axis enhances tumorigenicity. Through an EZH2/PBX1 signaling pathway, lncRNA NEAT1 facilitating immune evasion in multiple myeloma cells (Marin-Muller et al., 2013; Wang QM. et al., 2024). MiR-650 is upregulated in *Helicobacter pylori* ^+^ tissues and cells, and inhibition of miR-650 attenuates cell proliferation, invasion, and migration, but enhances apoptosis. PBX1 is overexpressed and promotes miR-650 transcription in H. pylori-associated gastric carcinoma tissues and cells. Overexpression of PBX1 abolishes the effects of miR-650 inhibitors on gastric cancer cells (Liu et al., 2021).

Cancer cells overexpressing PBX1 exhibit enhanced growth, invasion, epithelial-mesenchymal transition (EMT), and cisplatin resistance, whereas PBX1 silencing exhibits the opposite effect. The Phe252 hydrophobic residue in the first helix of the TALE homology domain of PBX1 is responsible for EMT, migration, and invasiveness. PBX1-interacting proteins increase cancer cell proliferation/M cell cycle transition through the activation of G1/S and G2, which is accompanied by an increase in the levels of positive cell cycle regulators, such as cyclin D1, cyclin A, and cyclin B. PBX1 interacts with estrogen receptor (ER) and its overexpression partially alleviates the inhibitory effect of ER antagonists on tumor cells and aggravates the tumor-promoting effect of estrogen on cancer cells (Magnani et al., 2013; Feng et al., 2015; He et al., 2017; Zhao et al., 2022). PBX1 expression is high in malignant hemangiomas and upregulates the expression of VEGF-A, base bFGF, flt-1 and flk-1 (the receptors of VEGF-A), and FGFR-1 (Kodama et al., 2009a; Kodama et al., 2009b). This may lead to a poor prognosis of the tumor because the ingrowth of blood vessels provides rich nutrients for the proliferation of tumor cells. Expression profiling and knockdown experiments in patients with Hodgkin lymphoma and cell lines have revealed that TLX2 is a target gene activated by PBX1. TBX15, which has anti-apoptotic effects, can be activated by TLX2 (Nagel et al., 2021). This may be one of the mechanisms whereby PBX1-related tumor cells escape death, and may be a new therapeutic target. Radiation resistance in oesophageal squamous cell carcinoma (OSCC) is a key factor leading to poor patient prognosis. The downregulation of PBX1 inhibits the expression of STAT3 and p-STAT3. The downregulation of PBX1 increases the radiosensitivity of oral squamous cell carcinoma cells and xenograft tumors through the PBX1/STAT3 pathway (Yu et al., 2020).

Thus, development of small molecule inhibitors targeting PBX1 transcriptional signaling could serve as a novel therapeutic strategy for PBX1-associated cancers (Shen et al., 2021). Interestingly, PBX1 does not always play an oncogenic role in tumors. It has been reported that PBX1 was inhibited in colorectal cancer. Mechanistically, PBX1 can inhibit the expression of Double cortin domain-containing protein 2 (DCDC2) and suppress spindle function. At the same time, the PBX1-DCDC2 axis controls the Wnt pathway in CRC cells. The overexpression of DCDC2 restores the proliferation, migration capabilities, and Wnt pathway of colorectal cancer cells (CRC). Overall, this study indicates that PBX1, as a transcription factor, inhibits DCDC2 expression and suppresses cell proliferation and migration in CRC by disrupting spindle function and the Wnt pathway (Martinou et al., 2022; Dai et al., 2023). A similar situation has also been found in non-small cell lung cancer (NSCLC). PBX1 is downregulated in NSCLC tissues and inhibits the proliferation and migration of NSCLC cells. Yuening Sun and colleagues discovered that PBX1 can bind to TRIM26 and undergo ubiquitination mediated by TRIM26, leading to proteasomal degradation. TRIM26 can inhibit the transcriptional activity of PBX1 and suppress its downstream gene RNF6, thereby promoting the proliferation and migration of NSCLC (Sun et al., 2023). Another research has revealed that PBX1-specific siRNA fosters an increase in the proliferation of these cells, while an overexpression of PBX1 conversely impedes their proliferation and inhibits DNA synthesis. These findings collectively emphasize PBX1’s role as a suppressor of lung cancer cell proliferation (Li et al., 2014b). The downregulation of M- and N-cadherin expression is considered to be related to the migration and invasion ability of tumor cells, and knockdown of *pbx1* markedly downregulates the expression of M-cadherin but upregulates N-cadherin expression (Thuault et al., 2013; Lin et al., 2021), which illustrates the complexity of PBX1 in tumor development.

## 4 Conclusion

Here, we have described the structure of PBX1 and its variant E2A-PBX1 as well as their roles in development or tumorigenesis, and have shown that their structure determines their functions. In summary, PBX1 and its variant E2A-PBX1 act as transcription factors, partnering with other proteins like HOX, Meis, and PREP to regulate gene expression. This regulation is vital for the maintenance and differentiation of various cell types. PBX1 plays a crucial role in controlling cell proliferation and differentiation, particularly in hematopoietic lineages, ensuring proper cell cycle progression and development. Both PBX1 and E2A-PBX1 have been implicated in cancer, where they may drive oncogenic processes through aberrant gene regulation and interactions with oncogenic partners. These interactions can induce the development of highly malignant solid and blood tumors that are difficult to treat, by stimulating a variety of targets in cooperation with co-factors, which induce the development of highly malignant solid and blood tumors that are difficult to treat by stimulating a variety of targets in cooperation with co-factors. In the future, researches on PBX1 and its variant will develop in the following directions: 1) Elucidation of mechanistic pathways: Further research is needed to fully understand the specific mechanisms by which PBX1 and its variants regulate different cellular processes. This includes identifying all interaction partners and target genes. 2) Role in disease progression: Investigating the role of PBX1 variants in disease, especially cancer, could reveal novel therapeutic targets. 3) Understanding how PBX1 contributes to tumorigenesis and metastasis could lead to new treatment strategies. 4) Therapeutic potential: Exploring PBX1 as a therapeutic target, either by modulating its activity or by targeting its downstream pathways, holds promise for treating diseases associated with its dysregulation. We believe that as research progresses and understanding of the oncogenic mechanisms of PBX1 and E2A-PBX1 deepens, the development of specific drugs against their targets would be key to treating their related cancers. The therapeutic effects of some small molecule drugs against the targets have also been verified, but there is still more work to be done. More and better drugs are needed. Based on the findings, we strongly recommend E2A-PBX1 as one of the genes for prenatal screening to reduce the incidence of childhood hematological malignancies.

## References

[B1] Agarwal S. Lakoma A. Chen Z. Hicks J. Metelitsa L. S. Kim E. S. (2015). G-CSF promotes neuroblastoma tumorigenicity and metastasis via STAT3-dependent cancer stem cell activation. Cancer Res. 75 (12), 2566–2579. 10.1158/0008-5472.Can-14-2946 25908586 PMC4470771

[B2] Aït Ghezali L. Arbabian A. Roudot H. Brouland J. P. Baran-Marszak F. Salvaris E. (2017). Induction of endoplasmic reticulum calcium pump expression during early leukemic B cell differentiation. J. Exp. Clin. Cancer Res. 36 (1), 87. 10.1186/s13046-017-0556-7 28651627 PMC5485704

[B3] Alankarage D. Szot J. O. Pachter N. Slavotinek A. Selleri L. Shieh J. T. (2020). Functional characterization of a novel PBX1 *de novo* missense variant identified in a patient with syndromic congenital heart disease. Hum. Mol. Genet. 29 (7), 1068–1082. 10.1093/hmg/ddz231 31625560 PMC7206850

[B4] Al-Shamsi B. Al-Kasbi G. Al-Kindi A. Bruwer Z. Al-Kharusi K. Al-Maawali A. (2022). Biallelic loss-of-function variants of GFRA1 cause lethal bilateral renal agenesis. Eur. J. Med. Genet. 65 (1), 104376. 10.1016/j.ejmg.2021.104376 34737117

[B5] Ariki R. Morikawa S. Mabuchi Y. Suzuki S. Nakatake M. Yoshioka K. (2014). Homeodomain transcription factor Meis1 is a critical regulator of adult bone marrow hematopoiesis. PLoS One 9 (2), e87646. 10.1371/journal.pone.0087646 24498346 PMC3911998

[B6] Arnovitz S. Mathur P. Tracy M. Mohsin A. Mondal S. Quandt J. (2022). Tcf-1 promotes genomic instability and T cell transformation in response to aberrant β-catenin activation. Proc. Natl. Acad. Sci. U. S. A. 119 (32), e2201493119. 10.1073/pnas.2201493119 35921443 PMC9371646

[B7] Aronheim A. Shiran R. Rosen A. Walker M. D. (1993). The E2A gene product contains two separable and functionally distinct transcription activation domains. Proc. Natl. Acad. Sci. U. S. A. 90 (17), 8063–8067. 10.1073/pnas.90.17.8063 8367464 PMC47288

[B8] Baba Y. Kurosaki T. (2011). Impact of Ca2+ signaling on B cell function. Trends Immunol. 32 (12), 589–594. 10.1016/j.it.2011.09.004 22000665

[B9] Bayly R. Murase T. Hyndman B. D. Savage R. Nurmohamed S. Munro K. (2006). Critical role for a single leucine residue in leukemia induction by E2A-PBX1. Mol. Cell Biol. 26 (17), 6442–6452. 10.1128/mcb.02025-05 16914730 PMC1592826

[B10] Belle I. Zhuang Y. (2014). E proteins in lymphocyte development and lymphoid diseases. Curr. Top. Dev. Biol. 110, 153–187. 10.1016/b978-0-12-405943-6.00004-x 25248476 PMC6504980

[B11] Bellomo G. Rainer C. Quaranta V. Astuti Y. Raymant M. Boyd E. (2022). Chemotherapy-induced infiltration of neutrophils promotes pancreatic cancer metastasis via Gas6/AXL signalling axis. Gut 71 (11), 2284–2299. 10.1136/gutjnl-2021-325272 35022267 PMC9554050

[B12] Bijl J. Krosl J. Lebert-Ghali C. E. Vacher J. Mayotte N. Sauvageau G. (2008). Evidence for Hox and E2A-PBX1 collaboration in mouse T-cell leukemia. Oncogene 27 (49), 6356–6364. 10.1038/onc.2008.233 18679416

[B13] Blasi F. Bruckmann C. Penkov D. Dardaei L. (2017). A tale of TALE, PREP1, PBX1, and MEIS1: interconnections and competition in cancer. Bioessays 39 (5). 10.1002/bies.201600245 28322463

[B14] Bobola N. Sagerström C. G. (2024). TALE transcription factors: cofactors no more. Semin. Cell Dev. Biol. 152-153, 76–84. 10.1016/j.semcdb.2022.11.015 36509674

[B15] Brendolan A. Ferretti E. Salsi V. Moses K. Quaggin S. Blasi F. (2005). A Pbx1-dependent genetic and transcriptional network regulates spleen ontogeny. Development 132 (13), 3113–3126. 10.1242/dev.01884 15944191

[B16] Bruckmann C. Tamburri S. De Lorenzi V. Doti N. Monti A. Mathiasen L. (2020). Mapping the native interaction surfaces of PREP1 with PBX1 by cross-linking mass-spectrometry and mutagenesis. Sci. Rep. 10 (1), 16809. 10.1038/s41598-020-74032-w 33033354 PMC7545097

[B17] Cai L. Y. Chen S. J. Xiao S. H. Sun Q. J. Ding C. H. Zheng B. N. (2021). Targeting p300/CBP attenuates hepatocellular carcinoma progression through epigenetic regulation of metabolism. Cancer Res. 81 (4), 860–872. 10.1158/0008-5472.Can-20-1323 33361394

[B18] Calvo K. R. Knoepfler P. McGrath S. Kamps M. P. (1999). An inhibitory switch derepressed by pbx, hox, and Meis/Prep1 partners regulates DNA-binding by pbx1 and E2a-pbx1 and is dispensable for myeloid immortalization by E2a-pbx1. Oncogene 18 (56), 8033–8043. 10.1038/sj.onc.1203377 10637514

[B19] Capellini T. D. Di Giacomo G. Salsi V. Brendolan A. Ferretti E. Srivastava D. (2006). Pbx1/Pbx2 requirement for distal limb patterning is mediated by the hierarchical control of Hox gene spatial distribution and Shh expression. Development 133 (11), 2263–2273. 10.1242/dev.02395 16672333

[B20] Capellini T. D. Zewdu R. Di Giacomo G. Asciutti S. Kugler J. E. Di Gregorio A. (2008). Pbx1/Pbx2 govern axial skeletal development by controlling Polycomb and Hox in mesoderm and Pax1/Pax9 in sclerotome. Dev. Biol. 321 (2), 500–514. 10.1016/j.ydbio.2008.04.005 18691704 PMC5918304

[B21] Chang C. P. de Vivo I. Cleary M. L. (1997). The Hox cooperativity motif of the chimeric oncoprotein E2a-Pbx1 is necessary and sufficient for oncogenesis. Mol. Cell Biol. 17 (1), 81–88. 10.1128/mcb.17.1.81 8972188 PMC231732

[B22] Chang C. P. Stankunas K. Shang C. Kao S. C. Twu K. Y. Cleary M. L. (2008). Pbx1 functions in distinct regulatory networks to pattern the great arteries and cardiac outflow tract. Development 135 (21), 3577–3586. 10.1242/dev.022350 18849531 PMC2680673

[B23] Charboneau A. East L. Mulholland N. Rohde M. Boudreau N. (2005). Pbx1 is required for Hox D3-mediated angiogenesis. Angiogenesis 8 (4), 289–296. 10.1007/s10456-005-9016-7 16328158

[B24] Chen C. C. Chen B. R. Wang Y. Curman P. Beilinson H. A. Brecht R. M. (2021). Sarco/endoplasmic reticulum Ca2+-ATPase (SERCA) activity is required for V(D)J recombination. J. Exp. Med. 218 (8), e20201708. 10.1084/jem.20201708 34033676 PMC8155808

[B25] Chen J. L. Li J. Kiriluk K. J. Rosen A. M. Paner G. P. Antic T. (2012). Deregulation of a Hox protein regulatory network spanning prostate cancer initiation and progression. Clin. Cancer Res. 18 (16), 4291–4302. 10.1158/1078-0432.Ccr-12-0373 22723371 PMC3479663

[B26] Chen J. S. Huang J. Q. Luo B. Dong S. H. Wang R. C. Jiang Z. K. (2019). PIK3CD induces cell growth and invasion by activating AKT/GSK-3β/β-catenin signaling in colorectal cancer. Cancer Sci. 110 (3), 997–1011. 10.1111/cas.13931 30618098 PMC6398891

[B27] Cheung C. L. Chan B. Y. Chan V. Ikegawa S. Kou I. Ngai H. (2009). Pre-B-cell leukemia homeobox 1 (PBX1) shows functional and possible genetic association with bone mineral density variation. Hum. Mol. Genet. 18 (4), 679–687. 10.1093/hmg/ddn397 19064610

[B28] Choi S. C. Park Y. P. Roach T. Jimenez D. Fisher A. Zadeh M. (2024). Lupus susceptibility gene Pbx1 controls the development, stability, and function of regulatory T cells via Rtkn2 expression. Sci. Adv. 10 (13), eadi4310. 10.1126/sciadv.adi4310 38536923 PMC10971436

[B29] Crisafulli L. Brindisi M. Liturri M. G. Sobacchi C. Ficara F. (2024). PBX1: a TALE of two seasons-key roles during development and in cancer. Front. Cell Dev. Biol. 12, 1372873. 10.3389/fcell.2024.1372873 38404687 PMC10884236

[B30] Crisafulli L. Muggeo S. Uva P. Wang Y. Iwasaki M. Locatelli S. (2019). MicroRNA-127-3p controls murine hematopoietic stem cell maintenance by limiting differentiation. Haematologica 104 (9), 1744–1755. 10.3324/haematol.2018.198499 30792210 PMC6717575

[B31] Cullmann K. Jahn M. Spindler M. Schenk F. Manukjan G. Mucci A. (2021). Forming megakaryocytes from murine-induced pluripotent stem cells by the inducible overexpression of supporting factors. Res. Pract. Thromb. Haemost. 5 (1), 111–124. 10.1002/rth2.12453 33537535 PMC7845061

[B32] Dai P. Li J. Chen Y. Zhang L. Zhang X. Wang J. (2021). Novel functional genes involved in transdifferentiation of canine ADMSCs into insulin-producing cells, as determined by absolute quantitative transcriptome sequencing analysis. Front. Cell Dev. Biol. 9, 685494. 10.3389/fcell.2021.685494 34262902 PMC8273515

[B33] Dai P. Qi G. Xu H. Zhu M. Li J. Chen Y. (2022). Reprogramming adipose mesenchymal stem cells into islet β-cells for the treatment of canine diabetes mellitus. Stem Cell Res. Ther. 13 (1), 370. 10.1186/s13287-022-03020-w 35902971 PMC9331803

[B34] Dai W. Liu Y. Zhang T. Huang Z. Xu X. Zhao Z. (2023). Spindle function and Wnt pathway inhibition by PBX1 to suppress tumor progression via downregulating DCDC2 in colorectal cancer. Oncogenesis 12 (1), 3. 10.1038/s41389-023-00448-4 36739270 PMC9899229

[B35] Danso-Abeam D. Staats K. A. Franckaert D. Van Den Bosch L. Liston A. Gray D. H. (2013). Aire mediates thymic expression and tolerance of pancreatic antigens via an unconventional transcriptional mechanism. Eur. J. Immunol. 43 (1), 75–84. 10.1002/eji.201242761 23041971

[B36] Dedera D. A. Waller E. K. LeBrun D. P. Sen-Majumdar A. Stevens M. E. Barsh G. S. (1993). Chimeric homeobox gene E2A-PBX1 induces proliferation, apoptosis, and malignant lymphomas in transgenic mice. Cell 74 (5), 833–843. 10.1016/0092-8674(93)90463-z 8104101

[B37] De Kumar B. Darland D. C. (2021). The Hox protein conundrum: the "specifics" of DNA binding for Hox proteins and their partners. Dev. Biol. 477, 284–292. 10.1016/j.ydbio.2021.06.002 34102167 PMC8846413

[B38] de Lau W. B. Hurenkamp J. Berendes P. Touw I. P. Clevers H. C. van Dijk M. A. (1998). The gene encoding the granulocyte colony-stimulating factor receptor is a target for deregulation in pre-B ALL by the t(1;19)-specific oncoprotein E2A-Pbx1. Oncogene 17 (4), 503–510. 10.1038/sj.onc.1201967 9696044

[B39] Denis C. M. Chitayat S. Plevin M. J. Wang F. Thompson P. Liu S. (2012). Structural basis of CBP/p300 recruitment in leukemia induction by E2A-PBX1. Blood 120 (19), 3968–3977. 10.1182/blood-2012-02-411397 22972988

[B40] Deucher A. M. Qi Z. Yu J. George T. I. Etzell J. E. (2015). BCL6 expression correlates with the t(1;19) translocation in B-lymphoblastic leukemia. Am. J. Clin. Pathol. 143 (4), 547–557. 10.1309/ajcpo4u4vyaaotel 25780007

[B41] Dhami K. Chakraborty A. Gururaja T. L. Cheung L. W. Sun C. DeAnda F. (2022). Kinase-deficient BTK mutants confer ibrutinib resistance through activation of the kinase HCK. Sci. Signal 15 (736), eabg5216. 10.1126/scisignal.abg5216 35639855

[B42] Dhawan S. Tschen S. I. Bhushan A. (2009). Bmi-1 regulates the Ink4a/Arf locus to control pancreatic beta-cell proliferation. Genes Dev. 23 (8), 906–911. 10.1101/gad.1742609 19390085 PMC2675870

[B43] Diakos C. Xiao Y. Zheng S. Kager L. Dworzak M. Wiemels J. L. (2014). Direct and indirect targets of the E2A-PBX1 leukemia-specific fusion protein. PLoS One 9 (2), e87602. 10.1371/journal.pone.0087602 24503810 PMC3913655

[B44] DiMartino J. F. Selleri L. Traver D. Firpo M. T. Rhee J. Warnke R. (2001). The Hox cofactor and proto-oncogene Pbx1 is required for maintenance of definitive hematopoiesis in the fetal liver. Blood 98 (3), 618–626. 10.1182/blood.v98.3.618 11468159

[B45] Duque-Afonso J. Feng J. Scherer F. Lin C. H. Wong S. H. Wang Z. (2015). Comparative genomics reveals multistep pathogenesis of E2A-PBX1 acute lymphoblastic leukemia. J. Clin. Invest. 125 (9), 3667–3680. 10.1172/jci81158 26301816 PMC4588292

[B46] Duque-Afonso J. Lin C. H. Han K. Wei M. C. Feng J. Kurzer J. H. (2016). E2A-PBX1 remodels oncogenic signaling networks in B-cell precursor acute lymphoid leukemia. Cancer Res. 76 (23), 6937–6949. 10.1158/0008-5472.Can-16-1899 27758892 PMC5634812

[B47] Dutta A. Yang Y. Le B. T. Zhang Y. Abdel-Wahab O. Zang C. (2021). U2af1 is required for survival and function of hematopoietic stem/progenitor cells. Leukemia 35 (8), 2382–2398. 10.1038/s41375-020-01116-x 33414485 PMC8283943

[B48] Eldfors S. Kuusanmäki H. Kontro M. Majumder M. M. Parsons A. Edgren H. (2017). Idelalisib sensitivity and mechanisms of disease progression in relapsed TCF3-PBX1 acute lymphoblastic leukemia. Leukemia 31 (1), 51–57. 10.1038/leu.2016.202 27461063 PMC5220125

[B49] Engwa G. A. Nweke F. N. Karngong G. N. Afiukwa C. A. Nwagu K. E. (2020). Understanding the pathogenesis, therapeutic targets/drug action and pharmacogenetics of type 2 diabetes: is there a future for personalised medicine? Endocr. Metab. Immune Disord. Drug Targets 20 (10), 1569–1589. 10.2174/1871530320666200425202312 32334506

[B50] Eyal S. Kult S. Rubin S. Krief S. Felsenthal N. Pineault K. M. (2019). Bone morphology is regulated modularly by global and regional genetic programs. Development 146 (14), dev167882. 10.1242/dev.167882 31221640 PMC6679367

[B51] Familiades J. Bousquet M. Lafage-Pochitaloff M. Béné M. C. Beldjord K. De Vos J. (2009). PAX5 mutations occur frequently in adult B-cell progenitor acute lymphoblastic leukemia and PAX5 haploinsufficiency is associated with BCR-ABL1 and TCF3-PBX1 fusion genes: a GRAALL study. Leukemia 23 (11), 1989–1998. 10.1038/leu.2009.135 19587702

[B52] Fang C. H. Lin Y. T. Liang C. M. Liang S. M. (2020). A novel c-Kit/phospho-prohibitin axis enhances ovarian cancer stemness and chemoresistance via Notch3-PBX1 and β-catenin-ABCG2 signaling. J. Biomed. Sci. 27 (1), 42. 10.1186/s12929-020-00638-x 32169072 PMC7071647

[B53] Farber P. J. Mittermaier A. (2011). Concerted dynamics link allosteric sites in the PBX homeodomain. J. Mol. Biol. 405 (3), 819–830. 10.1016/j.jmb.2010.11.016 21087615

[B54] Feldman B. J. Reid T. R. Cleary M. L. (1997). Pim1 cooperates with E2a-Pbx1 to facilitate the progression of thymic lymphomas in transgenic mice. Oncogene 15 (22), 2735–2742. 10.1038/sj.onc.1201670 9401000

[B55] Feng Y. Li L. Zhang X. Zhang Y. Liang Y. Lv J. (2015). Hematopoietic pre-B cell leukemia transcription factor interacting protein is overexpressed in gastric cancer and promotes gastric cancer cell proliferation, migration, and invasion. Cancer Sci. 106 (10), 1313–1322. 10.1111/cas.12754 26211905 PMC4638003

[B56] Feske S. Skolnik E. Y. Prakriya M. (2012). Ion channels and transporters in lymphocyte function and immunity. Nat. Rev. Immunol. 12 (7), 532–547. 10.1038/nri3233 22699833 PMC3670817

[B57] Ficara F. Crisafulli L. Lin C. Iwasaki M. Smith K. S. Zammataro L. (2013). Pbx1 restrains myeloid maturation while preserving lymphoid potential in hematopoietic progenitors. J. Cell Sci. 126 (Pt 14), 3181–3191. 10.1242/jcs.125435 23660001 PMC3711206

[B58] Ficara F. Murphy M. J. Lin M. Cleary M. L. (2008). Pbx1 regulates self-renewal of long-term hematopoietic stem cells by maintaining their quiescence. Cell Stem Cell 2 (5), 484–496. 10.1016/j.stem.2008.03.004 18462698 PMC2416441

[B59] Figueiredo M. Zilhão R. Neves H. (2020). Thymus inception: molecular network in the early stages of thymus organogenesis. Int. J. Mol. Sci. 21 (16), 5765. 10.3390/ijms21165765 32796710 PMC7460828

[B60] Fischbach N. A. Rozenfeld S. Shen W. Fong S. Chrobak D. Ginzinger D. (2005). HOXB6 overexpression in murine bone marrow immortalizes a myelomonocytic precursor *in vitro* and causes hematopoietic stem cell expansion and acute myeloid leukemia *in vivo* . Blood 105 (4), 1456–1466. 10.1182/blood-2004-04-1583 15522959

[B61] Francis J. C. Gardiner J. R. Renaud Y. Chauhan R. Weinstein Y. Gomez-Sanchez C. (2021). HOX genes promote cell proliferation and are potential therapeutic targets in adrenocortical tumours. Br. J. Cancer 124 (4), 805–816. 10.1038/s41416-020-01166-z 33214683 PMC7884796

[B62] Francois M. Donovan P. Fontaine F. (2020). Modulating transcription factor activity: interfering with protein-protein interaction networks. Semin. Cell Dev. Biol. 99, 12–19. 10.1016/j.semcdb.2018.07.019 30172762

[B63] Fu J. Bian M. Jiang Q. Zhang C. (2007). Roles of Aurora kinases in mitosis and tumorigenesis. Mol. Cancer Res. 5 (1), 1–10. 10.1158/1541-7786.Mcr-06-0208 17259342

[B64] Fu X. Roberts W. G. Nobile V. Shapiro R. Kamps M. P. (1999). mAngiogenin-3, a target gene of oncoprotein E2a-Pbx1, encodes a new angiogenic member of the angiogenin family. Growth factors. 17 (2), 125–137. 10.3109/08977199909103521 10595312

[B65] Gao H. Cao M. Deng K. Yang Y. Song J. Ni M. (2022). The lineage differentiation and dynamic heterogeneity of thymic epithelial cells during thymus organogenesis. Front. Immunol. 13, 805451. 10.3389/fimmu.2022.805451 35273595 PMC8901506

[B66] Gordon J. A. Hassan M. Q. Koss M. Montecino M. Selleri L. van Wijnen A. J. (2011). Epigenetic regulation of early osteogenesis and mineralized tissue formation by a HOXA10-PBX1-associated complex. Cells Tissues Organs 194 (2-4), 146–150. 10.1159/000324790 21597276 PMC3178072

[B67] Gordon J. A. Hassan M. Q. Saini S. Montecino M. van Wijnen A. J. Stein G. S. (2010). Pbx1 represses osteoblastogenesis by blocking Hoxa10-mediated recruitment of chromatin remodeling factors. Mol. Cell Biol. 30 (14), 3531–3541. 10.1128/mcb.00889-09 20439491 PMC2897555

[B68] Grebbin B. M. Hau A. C. Groß A. Anders-Maurer M. Schramm J. Koss M. (2016). Pbx1 is required for adult subventricular zone neurogenesis. Development 143 (13), 2281–2291. 10.1242/dev.128033 27226325 PMC4958316

[B69] Grebbin B. M. Schulte D. (2017). PBX1 as pioneer factor: a case still open. Front. Cell Dev. Biol. 5, 9. 10.3389/fcell.2017.00009 28261581 PMC5306212

[B70] Gregory G. D. Miccio A. Bersenev A. Wang Y. Hong W. Zhang Z. (2010). FOG1 requires NuRD to promote hematopoiesis and maintain lineage fidelity within the megakaryocytic-erythroid compartment. Blood 115 (11), 2156–2166. 10.1182/blood-2009-10-251280 20065294 PMC2844012

[B71] Grüninger P. K. Uhl F. Herzog H. Gentile G. Andrade-Martinez M. Schmidt T. (2022). Functional characterization of the PI3K/AKT/MTOR signaling pathway for targeted therapy in B-precursor acute lymphoblastic leukemia. Cancer Gene Ther. 29 (11), 1751–1760. 10.1038/s41417-022-00491-0 35794338 PMC9663301

[B72] Gu S. Zhang J. Han X. Ding H. Yao C. Ye Z. (2023). Involvement of transcriptional factor Pbx1 in peripheral B cell homeostasis to constrain lupus autoimmunity. Arthritis Rheumatol. 75 (8), 1381–1394. 10.1002/art.42487 36862399

[B73] Gulotta M. R. De Simone G. John J. Perricone U. Brancale A. (2021). A computer-based methodology to design non-standard peptides potentially able to prevent HOX-PBX1-associated cancer diseases. Int. J. Mol. Sci. 22 (11), 5670. 10.3390/ijms22115670 34073517 PMC8198631

[B74] Guo H. Ma O. Speck N. A. Friedman A. D. (2012). Runx1 deletion or dominant inhibition reduces Cebpa transcription via conserved promoter and distal enhancer sites to favor monopoiesis over granulopoiesis. Blood 119 (19), 4408–4418. 10.1182/blood-2011-12-397091 22451420 PMC3362359

[B75] Guo J. Liu Y. Lv J. Zou B. Chen Z. Li K. (2021). BCL6 confers KRAS-mutant non-small-cell lung cancer resistance to BET inhibitors. J. Clin. Invest. 131 (1), e133090. 10.1172/jci133090 33393503 PMC7773368

[B76] Hanley O. Zewdu R. Cohen L. J. Jung H. Lacombe J. Philippidou P. (2016). Parallel pbx-dependent pathways govern the coalescence and fate of motor columns. Neuron 91 (5), 1005–1020. 10.1016/j.neuron.2016.07.043 27568519 PMC5017921

[B77] Hao Y. Zong X. Ren P. Qian Y. Fu A. (2021). Basic helix-loop-helix (bHLH) transcription factors regulate a wide range of functions in arabidopsis. Int. J. Mol. Sci. 22 (13), 7152. 10.3390/ijms22137152 34281206 PMC8267941

[B78] Hartsink-Segers S. A. Zwaan C. M. Exalto C. Luijendijk M. W. Calvert V. S. Petricoin E. F. (2013). Aurora kinases in childhood acute leukemia: the promise of aurora B as therapeutic target. Leukemia 27 (3), 560–568. 10.1038/leu.2012.256 22940834 PMC3593181

[B79] He C. Wang Z. Zhang L. Yang L. Li J. Chen X. (2017). A hydrophobic residue in the TALE homeodomain of PBX1 promotes epithelial-to-mesenchymal transition of gastric carcinoma. Oncotarget 8 (29), 46818–46833. 10.18632/oncotarget.17473 28514754 PMC5564525

[B80] Heidet L. Morinière V. Henry C. De Tomasi L. Reilly M. L. Humbert C. (2017). Targeted exome sequencing identifies PBX1 as involved in monogenic congenital anomalies of the kidney and urinary tract. J. Am. Soc. Nephrol. 28 (10), 2901–2914. 10.1681/asn.2017010043 28566479 PMC5619971

[B81] Herzig S. Fuzesi L. Knepel W. (2000). Heterodimeric Pbx-Prep1 homeodomain protein binding to the glucagon gene restricting transcription in a cell type-dependent manner. J. Biol. Chem. 275 (36), 27989–27999. 10.1074/jbc.M003345200 10869353

[B82] Holmlund T. Lindberg M. J. Grander D. Wallberg A. E. (2013). GCN5 acetylates and regulates the stability of the oncoprotein E2A-PBX1 in acute lymphoblastic leukemia. Leukemia 27 (3), 578–585. 10.1038/leu.2012.265 23044487

[B83] Hu J. Yang H. Wang X. Ding J. Liao P. Zhu G. (2023). A novel pathogenic variant c.262delA in PBX1 causing oligomeganephronia identified using whole-exome sequencing and a literature review. Am. J. Med. Genet. A 191 (12), 2850–2855. 10.1002/ajmg.a.63364 37571997

[B84] Hu K. Miao L. Goodwin T. J. Li J. Liu Q. Huang L. (2017). Quercetin remodels the tumor microenvironment to improve the permeation, retention, and antitumor effects of nanoparticles. ACS Nano 11 (5), 4916–4925. 10.1021/acsnano.7b01522 28414916 PMC5961944

[B85] Hunger S. P. Galili N. Carroll A. J. Crist W. M. Link M. P. Cleary M. L. (1991). The t(1;19)(q23;p13) results in consistent fusion of E2A and PBX1 coding sequences in acute lymphoblastic leukemias. Blood 77 (4), 687–693. 10.1182/blood.V77.4.687.687 1671560

[B86] Izraeli S. Henn T. Strobl H. Ludwig W. D. Kovar H. Haas O. A. (1993). Expression of identical E2A/PBX1 fusion transcripts occurs in both pre-B and early pre-B immunological subtypes of childhood acute lymphoblastic leukemia. Leukemia 7 (12), 2054–2056. PMID 8255105. 8255105

[B87] Izraeli S. Kovar H. Gadner H. Lion T. (1992). Unexpected heterogeneity in E2A/PBX1 fusion messenger RNA detected by the polymerase chain reaction in pediatric patients with acute lymphoblastic leukemia. Blood 80 (6), 1413–1417. 10.1182/blood.V80.6.1413.1413 1520867

[B88] Jacobs J. J. Kieboom K. Marino S. DePinho R. A. van Lohuizen M. (1999). The oncogene and Polycomb-group gene bmi-1 regulates cell proliferation and senescence through the ink4a locus. Nature 397 (6715), 164–168. 10.1038/16476 9923679

[B89] Jiang Y. Liu F. Zou F. Zhang Y. Wang B. Zhang Y. (2019). PBX homeobox 1 enhances hair follicle mesenchymal stem cell proliferation and reprogramming through activation of the AKT/glycogen synthase kinase signaling pathway and suppression of apoptosis. Stem Cell Res. Ther. 10 (1), 268. 10.1186/s13287-019-1382-y 31443676 PMC6708256

[B90] Jing H. Reed A. Ulanovskaya O. A. Grigoleit J. S. Herbst D. M. Henry C. L. (2021). Phospholipase Cγ2 regulates endocannabinoid and eicosanoid networks in innate immune cells. Proc. Natl. Acad. Sci. U. S. A. 118 (41), e2112971118. 10.1073/pnas.2112971118 34607960 PMC8522274

[B91] Jurberg A. D. Vasconcelos-Fontes L. Cotta-de-Almeida V. (2015). A tale from TGF-β superfamily for thymus ontogeny and function. Front. Immunol. 6, 442. 10.3389/fimmu.2015.00442 26441956 PMC4564722

[B92] Kamps M. P. Murre C. Sun X. H. Baltimore D. (1990). A new homeobox gene contributes the DNA binding domain of the t(1;19) translocation protein in pre-B ALL. Cell 60 (4), 547–555. 10.1016/0092-8674(90)90658-2 1967983

[B93] Kao T. W. Chen H. H. Lin J. Wang T. L. Shen Y. A. (2024). PBX1 as a novel master regulator in cancer: its regulation, molecular biology, and therapeutic applications. Biochim. Biophys. Acta Rev. Cancer 1879 (2), 189085. 10.1016/j.bbcan.2024.189085 38341110

[B94] Karagiannidis I. Salataj E. Said Abu Egal E. Beswick E. J. (2021). G-CSF in tumors: aggressiveness, tumor microenvironment and immune cell regulation. Cytokine 142, 155479. 10.1016/j.cyto.2021.155479 33677228 PMC8044051

[B95] Khumukcham S. S. Manavathi B. (2021). Two decades of a protooncogene HPIP/PBXIP1: uncovering the tale from germ cell to cancer. Biochim. Biophys. Acta Rev. Cancer 1876 (1), 188576. 10.1016/j.bbcan.2021.188576 34090932

[B96] Kia F. Sarafoglou K. Mooganayakanakote Siddappa A. Roberts K. D. (2019). Partial gonadal dysgenesis associated with a pathogenic variant of PBX1 transcription factor. BMJ Case Rep. 12 (7), e227986. 10.1136/bcr-2018-227986 PMC6626438 31302614

[B97] Kilstrup-Nielsen C. Alessio M. Zappavigna V. (2003). PBX1 nuclear export is regulated independently of PBX-MEINOX interaction by PKA phosphorylation of the PBC-B domain. Embo J. 22 (1), 89–99. 10.1093/emboj/cdg010 12505987 PMC140055

[B98] Kim S. K. MacDonald R. J. (2002). Signaling and transcriptional control of pancreatic organogenesis. Curr. Opin. Genet. Dev. 12 (5), 540–547. 10.1016/s0959-437x(02)00338-6 12200159

[B99] Kim S. K. Selleri L. Lee J. S. Zhang A. Y. Gu X. Jacobs Y. (2002). Pbx1 inactivation disrupts pancreas development and in Ipf1-deficient mice promotes diabetes mellitus. Nat. Genet. 30 (4), 430–435. 10.1038/ng860 11912494

[B100] Knoepfler P. S. Calvo K. R. Chen H. Antonarakis S. E. Kamps M. P. (1997). Meis1 and pKnox1 bind DNA cooperatively with Pbx1 utilizing an interaction surface disrupted in oncoprotein E2a-Pbx1. Proc. Natl. Acad. Sci. U. S. A. 94 (26), 14553–14558. 10.1073/pnas.94.26.14553 9405651 PMC25052

[B101] Kodama A. Sakai H. Matsuura S. Murakami M. Murai A. Mori T. (2009b). Establishment of canine hemangiosarcoma xenograft models expressing endothelial growth factors, their receptors, and angiogenesis-associated homeobox genes. BMC Cancer 9, 363. 10.1186/1471-2407-9-363 19825192 PMC2768746

[B102] Kodama A. Sakai H. Murakami M. Murai A. Mori T. Maruo K. (2009a). Immunohistochemical demonstration of angiogenesis-associated homeobox proteins in canine vascular tumours. J. Comp. Pathol. 141 (2-3), 199–203. 10.1016/j.jcpa.2009.04.004 19505696

[B103] Kömüves L. G. Shen W. F. Kwong A. Stelnicki E. Rozenfeld S. Oda Y. (2000). Changes in HOXB6 homeodomain protein structure and localization during human epidermal development and differentiation. Dev. Dyn. 218 (4), 636–647. 10.1002/1097-0177(2000)9999:9999<::Aid-dvdy1014>3.0.Co;2-i 10906782

[B104] Koss M. Bolze A. Brendolan A. Saggese M. Capellini T. D. Bojilova E. (2012). Congenital asplenia in mice and humans with mutations in a Pbx/Nkx2-5/p15 module. Dev. Cell 22 (5), 913–926. 10.1016/j.devcel.2012.02.009 22560297 PMC3356505

[B105] LeBrun D. P. (2003). E2A basic helix-loop-helix transcription factors in human leukemia. Front. Biosci. 8, s206–s222. 10.2741/1030 12700034

[B106] Lee Y. L. Ito K. Pi W. C. Lin I. H. Chu C. S. Malik S. (2021). Mediator subunit MED1 is required for E2A-PBX1-mediated oncogenic transcription and leukemic cell growth. Proc. Natl. Acad. Sci. U. S. A. 118 (6), e1922864118. 10.1073/pnas.1922864118 33542097 PMC8017927

[B107] Le Tanno P. Breton J. Bidart M. Satre V. Harbuz R. Ray P. F. (2017). PBX1 haploinsufficiency leads to syndromic congenital anomalies of the kidney and urinary tract (CAKUT) in humans. J. Med. Genet. 54 (7), 502–510. 10.1136/jmedgenet-2016-104435 28270404

[B108] Li W. Huang K. Guo H. Cui G. Zhao S. (2014b). Inhibition of non-small-cell lung cancer cell proliferation by Pbx1. Chin. J. Cancer Res. 26 (5), 573–578. 10.3978/j.issn.1000-9604.2014.08.21 25400423 PMC4220252

[B109] Li W. Lin C. Y. Shang C. Han P. Xiong Y. Lin C. J. (2014a). Pbx1 activates Fgf10 in the mesenchyme of developing lungs. Genesis 52 (5), 399–407. 10.1002/dvg.22764 24591256

[B110] Licht J. D. (2020). Oncogenesis by E2A-PBX1 in ALL: RUNX and more. Blood 136 (1), 3–4. 10.1182/blood.2020005879 32614960

[B111] Lin C. H. Wang Z. Duque-Afonso J. Wong S. H. Demeter J. Loktev A. V. (2019). Oligomeric self-association contributes to E2A-PBX1-mediated oncogenesis. Sci. Rep. 9 (1), 4915. 10.1038/s41598-019-41393-w 30894657 PMC6426973

[B112] Lin C. H. Wong S. H. Kurzer J. H. Schneidawind C. Wei M. C. Duque-Afonso J. (2018). SETDB2 links e2a-PBX1 to cell-cycle dysregulation in acute leukemia through CDKN2C repression. Cell Rep. 23 (4), 1166–1177. 10.1016/j.celrep.2018.03.124 29694893 PMC5963704

[B113] Lin Y. J. Kao C. H. Hsiao S. P. Chen S. L. (2021). The cooperation of cis-elements during M-cadherin promoter activation. Biochem. J. 478 (4), 911–926. 10.1042/bcj20200535 33527978

[B114] Liu G. J. Cimmino L. Jude J. G. Hu Y. Witkowski M. T. McKenzie M. D. (2014). Pax5 loss imposes a reversible differentiation block in B-progenitor acute lymphoblastic leukemia. Genes Dev. 28 (12), 1337–1350. 10.1101/gad.240416.114 24939936 PMC4066403

[B115] Liu J. Wang L. Li J. Xu Y. (2021). Upregulation of microRNA-650 by PBX1 is correlated with the development of Helicobacter pylori-associated gastric carcinoma. Neoplasma 68 (2), 262–272. 10.4149/neo_2020_200806N823 33147052

[B116] Liu T. Branch D. R. Jin T. (2006). Pbx1 is a co-factor for Cdx-2 in regulating proglucagon gene expression in pancreatic A cells. Mol. Cell Endocrinol. 249 (1-2), 140–149. 10.1016/j.mce.2006.02.007 16574312

[B117] Liu Y. Feng J. Yuan K. Wu Z. Hu L. Lu Y. (2022a). The oncoprotein BCL6 enables solid tumor cells to evade genotoxic stress. Elife 11, e69255. 10.7554/eLife.69255 35503721 PMC9064299

[B118] Liu Y. Zhai E. Chen J. Qian Y. Zhao R. Ma Y. (2022b). m(6) A-mediated regulation of PBX1-GCH1 axis promotes gastric cancer proliferation and metastasis by elevating tetrahydrobiopterin levels. Cancer Commun. (Lond). 42 (4), 327–344. 10.1002/cac2.12281 35261206 PMC9017753

[B119] Longobardi E. Penkov D. Mateos D. De Florian G. Torres M. Blasi F. (2014). Biochemistry of the tale transcription factors PREP, MEIS, and PBX in vertebrates. Dev. Dyn. 243 (1), 59–75. 10.1002/dvdy.24016 23873833 PMC4232920

[B120] Losa M. Barozzi I. Osterwalder M. Hermosilla-Aguayo V. Morabito A. Chacón B. H. (2023). A spatio-temporally constrained gene regulatory network directed by PBX1/2 acquires limb patterning specificity via HAND2. Nat. Commun. 14 (1), 3993. 10.1038/s41467-023-39443-z 37414772 PMC10325989

[B121] Lu Q. Kamps M. P. (1996). Structural determinants within Pbx1 that mediate cooperative DNA binding with pentapeptide-containing Hox proteins: proposal for a model of a Pbx1-Hox-DNA complex. Mol. Cell Biol. 16 (4), 1632–1640. 10.1128/mcb.16.4.1632 8657138 PMC231149

[B122] Lu Q. Knoepfler P. S. Scheele J. Wright D. D. Kamps M. P. (1995). Both Pbx1 and E2A-Pbx1 bind the DNA motif ATCAATCAA cooperatively with the products of multiple murine Hox genes, some of which are themselves oncogenes. Mol. Cell Biol. 15 (7), 3786–3795. 10.1128/mcb.15.7.3786 7791786 PMC230617

[B123] Lüdtke T. H. Wojahn I. Kleppa M. J. Schierstaedt J. Christoffels V. M. Künzler P. (2021). Combined genomic and proteomic approaches reveal DNA binding sites and interaction partners of TBX2 in the developing lung. Respir. Res. 22 (1), 85. 10.1186/s12931-021-01679-y 33731112 PMC7968368

[B124] Ma J. Qin Y. Liu W. Duan H. Xia M. Chen Z. J. (2011). Analysis of PBX1 mutations in 192 Chinese women with Müllerian duct abnormalities. Fertil. Steril. 95 (8), 2615–2617. 10.1016/j.fertnstert.2011.04.074 21575942

[B125] Magnani L. Ballantyne E. B. Zhang X. Lupien M. (2011). PBX1 genomic pioneer function drives ERα signaling underlying progression in breast cancer. PLoS Genet. 7 (11), e1002368. 10.1371/journal.pgen.1002368 22125492 PMC3219601

[B126] Magnani L. Patten D. K. Nguyen V. T. Hong S. P. Steel J. H. Patel N. (2015). The pioneer factor PBX1 is a novel driver of metastatic progression in ERα-positive breast cancer. Oncotarget 6 (26), 21878–21891. 10.18632/oncotarget.4243 26215677 PMC4673133

[B127] Magnani L. Stoeck A. Zhang X. Lánczky A. Mirabella A. C. Wang T. L. (2013). Genome-wide reprogramming of the chromatin landscape underlies endocrine therapy resistance in breast cancer. Proc. Natl. Acad. Sci. U. S. A. 110 (16), E1490–E1499. 10.1073/pnas.1219992110 23576735 PMC3631697

[B128] Marin-Muller C. Li D. Bharadwaj U. Li M. Chen C. Hodges S. E. (2013). A tumorigenic factor interactome connected through tumor suppressor microRNA-198 in human pancreatic cancer. Clin. Cancer Res. 19 (21), 5901–5913. 10.1158/1078-0432.Ccr-12-3776 23989979 PMC3920728

[B129] Martinou E. G. Moller-Levet C. S. Angelidi A. M. (2022). PBX4 functions as a potential novel oncopromoter in colorectal cancer: a comprehensive analysis of the PBX gene family. Am. J. Cancer Res. 12 (2), 585–600. PMID: 35261789. 35261789 PMC8899996

[B130] Mary L. Leclerc D. Labalme A. Bellaud P. Mazaud-Guittot S. Dréano S. (2023). Functional assessment of a new PBX1 variant in a 46,XY fetus with severe syndromic difference of sexual development through CRISPR-cas9 gene editing. Genes (Basel) 14 (2), 273. 10.3390/genes14020273 36833200 PMC9956894

[B131] Mathiasen L. Bruckmann C. Pasqualato S. Blasi F. (2015). Purification and characterization of a DNA-binding recombinant PREP1:PBX1 complex. PLoS One 10 (4), e0125789. 10.1371/journal.pone.0125789 25856340 PMC4391845

[B132] McCarron M. J. Irla M. Sergé A. Soudja S. M. Marie J. C. (2019). Transforming Growth Factor-beta signaling in αβ thymocytes promotes negative selection. Nat. Commun. 10 (1), 5690. 10.1038/s41467-019-13456-z 31857584 PMC6923358

[B133] McCulley D. J. Wienhold M. D. Hines E. A. Hacker T. A. Rogers A. Pewowaruk R. J. (2018). PBX transcription factors drive pulmonary vascular adaptation to birth. J. Clin. Invest. 128 (2), 655–667. 10.1172/jci93395 29251627 PMC5785269

[B134] McWhirter J. R. Neuteboom S. T. Wancewicz E. V. Monia B. P. Downing J. R. Murre C. (1999). Oncogenic homeodomain transcription factor E2A-Pbx1 activates a novel WNT gene in pre-B acute lymphoblastoid leukemia. Proc. Natl. Acad. Sci. U. S. A. 96 (20), 11464–11469. 10.1073/pnas.96.20.11464 10500199 PMC18056

[B135] Miao Y. Tian L. Martin M. Paige S. L. Galdos F. X. Li J. (2020). Intrinsic endocardial defects contribute to hypoplastic left heart syndrome. Cell Stem Cell 27 (4), 574–589. 10.1016/j.stem.2020.07.015 32810435 PMC7541479

[B136] Migita N. A. Jotta P. Y. Nascimento N. P. D. Vasconcelos V. S. Centoducatte G. L. Massirer K. B. (2023). Classification and genetics of pediatric B-other acute lymphoblastic leukemia by targeted RNA sequencing. Blood Adv. 7 (13), 2957–2971. 10.1182/bloodadvances.2022009179 36848637 PMC10320209

[B137] Miyake N. Takahashi H. Nakamura K. Isidor B. Hiraki Y. Koshimizu E. (2020). Gain-of-Function MN1 truncation variants cause a recognizable syndrome with craniofacial and brain abnormalities. Am. J. Hum. Genet. 106 (1), 13–25. 10.1016/j.ajhg.2019.11.011 31839203 PMC7042485

[B138] Mo M. L. Chen Z. Zhou H. M. Li H. Hirata T. Jablons D. M. (2013). Detection of E2A-PBX1 fusion transcripts in human non-small-cell lung cancer. J. Exp. Clin. Cancer Res. 32 (1), 29. 10.1186/1756-9966-32-29 23688269 PMC3661382

[B139] Moisan V. Brousseau C. Tremblay J. J. (2022). Dynamic expression of the homeobox factor PBX1 during mouse testis development. Endocrines 3, 16–28. 10.3390/endocrines3010002

[B140] Mostufi-Zadeh-Haghighi G. Veratti P. Zodel K. Greve G. Waterhouse M. Zeiser R. (2023). Functional characterization of transforming growth factor-β signaling in dasatinib resistance and pre-BCR(+) acute lymphoblastic leukemia. Cancers (Basel) 15 (17), 4328. 10.3390/cancers15174328 37686604 PMC10486903

[B141] Muggeo S. Crisafulli L. Uva P. Fontana E. Ubezio M. Morenghi E. (2021). PBX1-directed stem cell transcriptional program drives tumor progression in myeloproliferative neoplasm. Stem Cell Rep. 16 (11), 2607–2616. 10.1016/j.stemcr.2021.09.016 PMC8581051 34678207

[B142] Murre C. (2019). Helix-loop-helix proteins and the advent of cellular diversity: 30 years of discovery. Genes Dev. 33 (1-2), 6–25. 10.1101/gad.320663.118 30602438 PMC6317319

[B143] Nagel S. Meyer C. (2022). Normal and aberrant TALE-class homeobox gene activities in pro-B-cells and B-cell precursor acute lymphoblastic leukemia. Int. J. Mol. Sci. 23 (19), 11874. 10.3390/ijms231911874 36233173 PMC9570312

[B144] Nagel S. Pommerenke C. Meyer C. MacLeod R. A. F. Drexler H. G. (2021). Establishment of the TALE-code reveals aberrantly activated homeobox gene PBX1 in Hodgkin lymphoma. PLoS One 16 (2), e0246603. 10.1371/journal.pone.0246603 33539429 PMC7861379

[B145] Nengroo M. A. Maheshwari S. Singh A. Verma A. Arya R. K. Chaturvedi P. (2021). CXCR4 intracellular protein promotes drug resistance and tumorigenic potential by inversely regulating the expression of Death Receptor 5. Cell Death Dis. 12 (5), 464. 10.1038/s41419-021-03730-8 33966046 PMC8106681

[B146] Nicosia L. Spencer G. J. Brooks N. Amaral F. M. R. Basma N. J. Chadwick J. A. (2023). Therapeutic targeting of EP300/CBP by bromodomain inhibition in hematologic malignancies. Cancer Cell 41 (12), 2136–2153.e13. 10.1016/j.ccell.2023.11.001 37995682

[B147] Nie L. Li Y. Xiao T. Zhang B. Zhao J. Hou W. (2022). A pathogenic variant of PBX1 identified by whole exome sequencing in a Chinese CAKUTHED case. Nephron 147, 311–316. 10.1159/000526847 36318887

[B148] Niu Y. Sengupta M. Titov A. A. Choi S. C. Morel L. (2017). The PBX1 lupus susceptibility gene regulates CD44 expression. Mol. Immunol. 85, 148–154. 10.1016/j.molimm.2017.02.016 28257976 PMC5389453

[B149] Okada Y. Nagai R. Sato T. Matsuura E. Minami T. Morita I. (2003). Homeodomain proteins MEIS1 and PBXs regulate the lineage-specific transcription of the platelet factor 4 gene. Blood 101 (12), 4748–4756. 10.1182/blood-2002-02-0380 12609849

[B150] Olatoke T. Wagner A. Astrinidis A. Zhang E. Y. Guo M. Zhang A. G. (2023). Single-cell multiomic analysis identifies a HOX-PBX gene network regulating the survival of lymphangioleiomyomatosis cells. Sci. Adv. 9 (19), eadf8549. 10.1126/sciadv.adf8549 37163604 PMC10171823

[B151] Oriente F. Cabaro S. Liotti A. Longo M. Parrillo L. Pagano T. B. (2013). PREP1 deficiency downregulates hepatic lipogenesis and attenuates steatohepatitis in mice. Diabetologia 56 (12), 2713–2722. 10.1007/s00125-013-3053-3 24052111

[B152] Oriente F. Perruolo G. Cimmino I. Cabaro S. Liotti A. Longo M. (2018). Prep1, A homeodomain transcription factor involved in glucose and lipid metabolism. Front. Endocrinol. (Lausanne) 9, 346. 10.3389/fendo.2018.00346 30002646 PMC6032887

[B153] Pan F. C. Wright C. (2011). Pancreas organogenesis: from bud to plexus to gland. Dev. Dyn. 240 (3), 530–565. 10.1002/dvdy.22584 21337462

[B154] Park J. T. Shih Ie M. Wang T. L. (2008). Identification of Pbx1, a potential oncogene, as a Notch3 target gene in ovarian cancer. Cancer Res. 68 (21), 8852–8860. 10.1158/0008-5472.Can-08-0517 18974129 PMC2636690

[B155] Penkov D. Mateos San Martín D. Fernandez-Díaz L. C. Rosselló C. A. Torroja C. Sánchez-Cabo F. (2013). Analysis of the DNA-binding profile and function of TALE homeoproteins reveals their specialization and specific interactions with Hox genes/proteins. Cell Rep. 3 (4), 1321–1333. 10.1016/j.celrep.2013.03.029 23602564

[B156] Penkov D. Palazzolo M. Mondino A. Blasi F. (2008). Cytosolic sequestration of Prep1 influences early stages of T cell development. PLoS One 3 (6), e2424. 10.1371/journal.pone.0002424 18560600 PMC2413408

[B157] Peters K. Panienka R. Li J. Klöppel G. Wang R. (2005). Expression of stem cell markers and transcription factors during the remodeling of the rat pancreas after duct ligation. Virchows Arch. 446 (1), 56–63. 10.1007/s00428-004-1145-7 15660282

[B158] Petzold F. Jin W. Hantmann E. Korbach K. Schönauer R. Halbritter J. (2022). Novel somatic PBX1 mosaicism likely masking syndromic CAKUT in an adult with bilateral kidney hypoplasia. Clin. Kidney J. 15 (7), 1333–1339. 10.1093/ckj/sfac092 35756743 PMC9217644

[B159] Pi W. C. Wang J. Shimada M. Lin J. W. Geng H. Lee Y. L. (2020). E2A-PBX1 functions as a coactivator for RUNX1 in acute lymphoblastic leukemia. Blood 136 (1), 11–23. 10.1182/blood.2019003312 32276273 PMC7332894

[B160] Pruitt S. C. Bussman A. Maslov A. Y. Natoli T. A. Heinaman R. (2004). Hox/Pbx and Brn binding sites mediate Pax3 expression *in vitro* and *in vivo* . Gene Expr. Patterns 4 (6), 671–685. 10.1016/j.modgep.2004.04.006 15465489

[B161] Qi J. C. Yang Z. Lin T. Ma L. Wang Y. X. Zhang Y. (2021). CDK13 upregulation-induced formation of the positive feedback loop among circCDK13, miR-212-5p/miR-449a and E2F5 contributes to prostate carcinogenesis. J. Exp. Clin. Cancer Res. 40 (1), 2. 10.1186/s13046-020-01814-5 33390186 PMC7780414

[B162] Reichlmeir M. Elias L. Schulte D. (2021). Posttranslational modifications in conserved transcription factors: a survey of the TALE-homeodomain superclass in human and mouse. Front. Cell Dev. Biol. 9, 648765. 10.3389/fcell.2021.648765 33768097 PMC7985065

[B163] Risolino M. Mandia N. Iavarone F. Dardaei L. Longobardi E. Fernandez S. (2014). Transcription factor PREP1 induces EMT and metastasis by controlling the TGF-β-SMAD3 pathway in non-small cell lung adenocarcinoma. Proc. Natl. Acad. Sci. U. S. A. 111 (36), E3775–E3784. 10.1073/pnas.1407074111 25157139 PMC4246949

[B164] Rothenberg E. V. (2022). Transcription factors specifically control change. Genes Dev. 36 (21-24), 1097–1099. 10.1101/gad.350308.122 36622807 PMC9851400

[B165] Sanyal M. Tung J. W. Karsunky H. Zeng H. Selleri L. Weissman I. L. (2007). B-cell development fails in the absence of the Pbx1 proto-oncogene. Blood 109 (10), 4191–4199. 10.1182/blood-2006-10-054213 17244677 PMC1885499

[B166] Scarlett C. J. Colvin E. K. Pinese M. Chang D. K. Morey A. L. Musgrove E. A. (2011). Recruitment and activation of pancreatic stellate cells from the bone marrow in pancreatic cancer: a model of tumor-host interaction. PLoS One 6 (10), e26088. 10.1371/journal.pone.0026088 22022519 PMC3193536

[B167] Schnabel C. A. Godin R. E. Cleary M. L. (2003a). Pbx1 regulates nephrogenesis and ureteric branching in the developing kidney. Dev. Biol. 254 (2), 262–276. 10.1016/s0012-1606(02)00038-6 12591246

[B168] Schnabel C. A. Selleri L. Cleary M. L. (2003b). Pbx1 is essential for adrenal development and urogenital differentiation. Genesis 37 (3), 123–130. 10.1002/gene.10235 14595835

[B169] Schwaller J. (2006). siRNA-mediated inhibition of E2A-PBX1 reduces EB-1 and Wnt16b expression in 697 pre-B leukemia cells. Haematologica 91 (6), 724. 10.3324/%x 16769568

[B170] Selleri L. DiMartino J. van Deursen J. Brendolan A. Sanyal M. Boon E. (2004). The TALE homeodomain protein Pbx2 is not essential for development and long-term survival. Mol. Cell Biol. 24 (12), 5324–5331. 10.1128/mcb.24.12.5324-5331.2004 15169896 PMC419882

[B171] Selleri L. Zappavigna V. Ferretti E. (2019). Building a perfect body’: control of vertebrate organogenesis by PBX-dependent regulatory networks. Genes Dev. 33 (5-6), 258–275. 10.1101/gad.318774.118 30824532 PMC6411007

[B172] Sgadò P. Ferretti E. Grbec D. Bozzi Y. Simon H. H. (2012). The atypical homeoprotein Pbx1a participates in the axonal pathfinding of mesencephalic dopaminergic neurons. Neural Dev. 7, 24. 10.1186/1749-8104-7-24 22748019 PMC3407702

[B173] Shen Y. A. Jung J. Shimberg G. D. Hsu F. C. Rahmanto Y. S. Gaillard S. L. (2021). Development of small molecule inhibitors targeting PBX1 transcription signaling as a novel cancer therapeutic strategy. iScience 24 (11), 103297. 10.1016/j.isci.2021.103297 34816098 PMC8591422

[B174] Shiozawa Y. Pedersen E. A. Taichman R. S. (2010). GAS6/Mer axis regulates the homing and survival of the E2A/PBX1-positive B-cell precursor acute lymphoblastic leukemia in the bone marrow niche. Exp. Hematol. 38 (2), 132–140. 10.1016/j.exphem.2009.11.002 19922767 PMC2815170

[B175] Shiraishi K. Yamasaki K. Nanba D. Inoue H. Hanakawa Y. Shirakata Y. (2007). Pre-B-cell leukemia transcription factor 1 is a major target of promyelocytic leukemia zinc-finger-mediated melanoma cell growth suppression. Oncogene 26 (3), 339–348. 10.1038/sj.onc.1209800 16862184

[B176] Sinclair P. B. Cranston R. E. Raninga P. Cheng J. Hanna R. Hawking Z. (2023). Disruption to the FOXO-PRDM1 axis resulting from deletions of chromosome 6 in acute lymphoblastic leukaemia. Leukemia 37 (3), 636–649. 10.1038/s41375-023-01816-0 36670235 PMC9991907

[B177] Slavotinek A. Risolino M. Losa M. Cho M. T. Monaghan K. G. Schneidman-Duhovny D. (2017). *De novo*, deleterious sequence variants that alter the transcriptional activity of the homeoprotein PBX1 are associated with intellectual disability and pleiotropic developmental defects. Hum. Mol. Genet. 26 (24), 4849–4860. 10.1093/hmg/ddx363 29036646 PMC6455034

[B178] Smith K. S. Chanda S. K. Lingbeek M. Ross D. T. Botstein D. van Lohuizen M. (2003). Bmi-1 regulation of INK4A-ARF is a downstream requirement for transformation of hematopoietic progenitors by E2a-Pbx1. Mol. Cell 12 (2), 393–400. 10.1016/s1097-2765(03)00277-6 14536079

[B179] Sui Y. Liu F. Zheng S. Liu X. Sun P. Yao C. (2023). G-quadruplexes folding mediates downregulation of PBX1 expression in melanoma. Signal Transduct. Target Ther. 8 (1), 12. 10.1038/s41392-022-01222-5 36604432 PMC9816092

[B180] Sun Y. Lin P. Zhou X. Ren Y. He Y. Liang J. (2023). TRIM26 promotes non-small cell lung cancer survival by inducing PBX1 degradation. Int. J. Biol. Sci. 19 (9), 2803–2816. 10.7150/ijbs.81726 37324936 PMC10266081

[B181] Sun Y. Zhu D. Chen F. Qian M. Wei H. Chen W. (2016). SFRP2 augments WNT16B signaling to promote therapeutic resistance in the damaged tumor microenvironment. Oncogene 35 (33), 4321–4334. 10.1038/onc.2015.494 26751775 PMC4994019

[B182] Sykes D. B. Kamps M. P. (2004). E2a/Pbx1 induces the rapid proliferation of stem cell factor-dependent murine pro-T cells that cause acute T-lymphoid or myeloid leukemias in mice. Mol. Cell Biol. 24 (3), 1256–1269. 10.1128/mcb.24.3.1256-1269.2004 14729970 PMC321418

[B183] Szekely B. Bossuyt V. Li X. Wali V. B. Patwardhan G. A. Frederick C. (2018). Immunological differences between primary and metastatic breast cancer. Ann. Oncol. 29 (11), 2232–2239. 10.1093/annonc/mdy399 30203045

[B184] Szot J. O. Cuny H. Blue G. M. Humphreys D. T. Ip E. Harrison K. (2018). A screening approach to identify clinically actionable variants causing congenital heart disease in exome data. Circ. Genom Precis. Med. 11 (3), e001978. 10.1161/circgen.117.001978 29555671

[B185] Tanaka M. Siemann D. W. (2020). Gas6/Axl signaling pathway in the tumor immune microenvironment. Cancers (Basel) 12 (7), 1850. 10.3390/cancers12071850 32660000 PMC7408754

[B186] Tang W. Zhou W. Xiang L. Wu X. Zhang P. Wang J. (2019). The p300/YY1/miR-500a-5p/HDAC2 signalling axis regulates cell proliferation in human colorectal cancer. Nat. Commun. 10 (1), 663. 10.1038/s41467-018-08225-3 30737378 PMC6368584

[B187] Thuault S. Hayashi S. Lagirand-Cantaloube J. Plutoni C. Comunale F. Delattre O. (2013). P-cadherin is a direct PAX3-FOXO1A target involved in alveolar rhabdomyosarcoma aggressiveness. Oncogene 32 (15), 1876–1887. 10.1038/onc.2012.217 22710718

[B188] Van Dijk M. A. Voorhoeve P. M. Murre C. (1993). Pbx1 is converted into a transcriptional activator upon acquiring the N-terminal region of E2A in pre-B-cell acute lymphoblastoid leukemia. Proc. Natl. Acad. Sci. U. S. A. 90 (13), 6061–6065. 10.1073/pnas.90.13.6061 8327485 PMC46867

[B189] Veiga R. N. de Oliveira J. C. Gradia D. F. (2021). PBX1: a key character of the hallmarks of cancer. J. Mol. Med. Berl. 99 (12), 1667–1680. 10.1007/s00109-021-02139-2 34529123

[B190] Villaescusa J. C. Li B. Toledo E. M. Rivetti di Val Cervo P. Yang S. Stott S. R. (2016). A PBX1 transcriptional network controls dopaminergic neuron development and is impaired in Parkinson’s disease. Embo J. 35 (18), 1963–1978. 10.15252/embj.201593725 27354364 PMC5282836

[B191] Wang B. Liu F. Liu Z. Han X. Lian A. Zhang Y. (2020b). Internalization of the TAT-PBX1 fusion protein significantly enhances the proliferation of human hair follicle-derived mesenchymal stem cells and delays their senescence. Biotechnol. Lett. 42 (10), 1877–1885. 10.1007/s10529-020-02909-x 32436118

[B192] Wang F. C. Zhang X. N. Wu S. X. He Z. Zhang L. Y. Yang Q. E. (2024a). Loss of PBX1 function in Leydig cells causes testicular dysgenesis and male sterility. Cell Mol. Life Sci. 81 (1), 212. 10.1007/s00018-024-05249-5 38724675 PMC11082031

[B193] Wang K. Rong L. Wei X. Zhang Q. (2020a). Analysis of antiapoptosis effect of netrin-1 on ischemic stroke and its molecular mechanism under deleted in colon cancer/extracellular signal-regulated kinase signaling pathway. Biomed. Res. Int. 2020, 8855949. 10.1155/2020/8855949 33274229 PMC7683118

[B194] Wang Q. M. Lian G. Y. Sheng S. M. Xu J. Ye L. L. Min C. (2024b). Exosomal lncRNA NEAT1 inhibits NK-cell activity to promote multiple myeloma cell immune escape via an EZH2/PBX1 Axis. Mol. Cancer Res. 22 (2), 125–136. 10.1158/1541-7786.Mcr-23-0282 37889101

[B195] Wang Y. Sui Y. Lian A. Han X. Liu F. Zuo K. (2021). PBX1 attenuates hair follicle-derived mesenchymal stem cell senescence and apoptosis by alleviating reactive oxygen species-mediated DNA damage instead of enhancing DNA damage repair. Front. Cell Dev. Biol. 9, 739868. 10.3389/fcell.2021.739868 34869323 PMC8634257

[B196] Wang Y. Sui Y. Niu Y. Liu D. Xu Q. Liu F. (2023). PBX1-SIRT1 positive feedback loop attenuates ROS-mediated HF-msc senescence and apoptosis. Stem Cell Rev. Rep. 19 (2), 443–454. 10.1007/s12015-022-10425-w 35962175 PMC9902417

[B197] Wang Z. Rice S. V. Chang T. C. Liu Y. Liu Q. Qin N. (2020c). Molecular mechanism of telomere length dynamics and its prognostic value in pediatric cancers. J. Natl. Cancer Inst. 112 (7), 756–764. 10.1093/jnci/djz210 31647544 PMC7357329

[B198] Wei X. Yu L. Li Y. (2018). PBX1 promotes the cell proliferation via JAK2/STAT3 signaling in clear cell renal carcinoma. Biochem. Biophys. Res. Commun. 500 (3), 650–657. 10.1016/j.bbrc.2018.04.127 29678569

[B199] Wu M. Xia X. Hu J. Fowlkes N. W. Li S. (2021). WSX1 act as a tumor suppressor in hepatocellular carcinoma by downregulating neoplastic PD-L1 expression. Nat. Commun. 12 (1), 3500. 10.1038/s41467-021-23864-9 34108491 PMC8190270

[B200] Xenou L. Papakonstanti E. A. (2020). p110δ PI3K as a therapeutic target of solid tumours. Clin. Sci. (Lond). 134 (12), 1377–1397. 10.1042/cs20190772 32556179

[B201] Xu B. Cai L. Butler J. M. Chen D. Lu X. Allison D. F. (2018). The chromatin remodeler BPTF activates a stemness gene-expression program essential for the maintenance of adult hematopoietic stem cells. Stem Cell Rep. 10 (3), 675–683. 10.1016/j.stemcr.2018.01.020 PMC5918338 29456179

[B202] Yu D. Ma Y. Feng C. Ma Z. Guo J. Chen H. (2020). PBX1 increases the radiosensitivity of oesophageal squamous cancer by targeting of STAT3. Pathol. Oncol. Res. 26 (4), 2161–2168. 10.1007/s12253-020-00803-5 32170580

[B203] Zdżalik-Bielecka D. Poświata A. Kozik K. Jastrzębski K. Schink K. O. Brewińska-Olchowik M. (2021). The GAS6-AXL signaling pathway triggers actin remodeling that drives membrane ruffling, macropinocytosis, and cancer-cell invasion. Proc. Natl. Acad. Sci. U. S. A. 118 (28), e2024596118. 10.1073/pnas.2024596118 34244439 PMC8285903

[B204] Zewdu R. Risolino M. Barbulescu A. Ramalingam P. Butler J. M. Selleri L. (2016). Spleen hypoplasia leads to abnormal stress hematopoiesis in mice with loss of Pbx homeoproteins in splenic mesenchyme. J. Anat. 229 (1), 153–169. 10.1111/joa.12479 27075259 PMC5341595

[B205] Zhang H. Wan Y. Wang H. Cai J. Yu J. Hu S. (2023b). Prognostic factors of childhood acute lymphoblastic leukemia with TCF3::PBX1 in CCCG-ALL-2015: a multicenter study. Cancer 129 (11), 1691–1703. 10.1002/cncr.34741 36943767

[B206] Zhang K. Liu D. Li Y. Shi Z. Guo J. Gao C. (2023a). The E3 ligase TRIM31 regulates hematopoietic stem cell homeostasis and MLL-AF9 leukemia. Haematologica 108 (8), 2116–2129. 10.3324/haematol.2022.281955 36632737 PMC10388288

[B207] Zhang X. Rowan S. Yue Y. Heaney S. Pan Y. Brendolan A. (2006). Pax6 is regulated by Meis and Pbx homeoproteins during pancreatic development. Dev. Biol. 300 (2), 748–757. 10.1016/j.ydbio.2006.06.030 17049510

[B208] Zhao Y. Che J. Tian A. Zhang G. Xu Y. Li S. (2022). PBX1 participates in estrogen-mediated bladder cancer progression and chemo-resistance affecting estrogen receptors. Curr. Cancer Drug Targets 22 (9), 757–770. 10.2174/1568009622666220413084456 35422219

[B209] Zhao Z. Dai X. Jiang G. Lin F. (2023). ASH2L controls ureteric bud morphogenesis through the regulation of RET/GFRA1 signaling activity in a mouse model. J. Am. Soc. Nephrol. 34 (6), 988–1002. 10.1681/asn.0000000000000099 36758123 PMC10278782

[B210] Zhou B. Chu X. Tian H. Liu T. Liu H. Gao W. (2021). The clinical outcomes and genomic landscapes of acute lymphoblastic leukemia patients with E2A-PBX1: a 10-year retrospective study. Am. J. Hematol. 96 (11), 1461–1471. 10.1002/ajh.26324 34406703

[B211] Zhou X. Zhang P. Liu N. Zhang X. Lv H. Xu W. (2023). Enhancing chemotherapy for pancreatic cancer through efficient and sustained tumor microenvironment remodeling with a fibroblast-targeted nanosystem. J. Control Release 361, 161–177. 10.1016/j.jconrel.2023.07.061 37536546

[B212] Zhou Y. Fu B. Xu X. Zhang J. Tong X. Wang Y. (2020). PBX1 expression in uterine natural killer cells drives fetal growth. Sci. Transl. Med. 12 (537), eaax1798. 10.1126/scitranslmed.aax1798 32238574

[B213] Zhu X. Wei L. Bai Y. Wu S. Han S. (2017). FoxC1 promotes epithelial-mesenchymal transition through PBX1 dependent transactivation of ZEB2 in esophageal cancer. Am. J. Cancer Res. 7 (8), 1642–1653. PMID:28861321. 28861321 PMC5574937

